# Neonatal Jaundice and Autism: Precautionary Principle Invocation Overdue

**DOI:** 10.7759/cureus.22512

**Published:** 2022-02-23

**Authors:** Vera K Wilde

**Affiliations:** 1 Medicine, Methods, Ethics, and Technology, Independent Researcher, Berlin, DEU

**Keywords:** neonatal feeding, autism spectrum disorder (asd), breastfeeding, neonatal jaundice, publication bias, statistical data analysis, neurodevelopment, starvation, preventive health, hyperbilirubinemia

## Abstract

Meta-analyses consistently find a substantial possible association between neonatal jaundice (hyperbilirubinemia) and later autism risk. The obvious question this poses is "what is the source of this risk?" This review explores the complementary roles of jaundice severity and time, racial and geographic disparities, and early infant feeding regime change, and discusses potential implications of these findings. A range of factors appears to increase the risk of autism development following neonatal jaundice, all of which are associated with the "exclusive breastfeeding" paradigm. Severity presents an intuitive risk factor in the context of bilirubin neurotoxicity; jaundice from the modal root cause of insufficient milk intake progresses as that condition persists. Racial and geographic disparities present another intuitive set of risk factors, including a heightened risk of missed diagnosis for darker-skinned neonates and delayed care access in poorer settings. In addition to these intuitive factors, near- or full-term as opposed to preterm status and phototherapy treatment may also heighten risk. These counter-intuitive findings provide additional support for deprivation/starvation as a crucial antecedent or independent variable, and time as a mediator to progression in and subsequent risk from jaundice; heightened medical monitoring and supplementation seem to protect preterms, and phototherapy risks iatrogenesis, having replaced without sufficient safety evidence the prior standard treatment of switching jaundiced, breastfed babies to formula. Critically, jaundice associated with insufficient milk intake due to breastfeeding insufficiencies is fully preventable and trivially treatable with appropriate supplemental milk. Feeding neonates adequately may play an important role in preventing autism and other neurodevelopmental disorders including attention deficit hyperactivity disorder, cerebral palsy, epilepsy, hearing impairment, learning disorders, and mood disorders. Precautionary principle invocation is overdue.

## Introduction and background

Recently, a third meta-analysis found neonatal jaundice is associated with substantial possible increased autism risk [1-3] - the latest in a series of repeated warnings of possible long-term neurodevelopmental harm from neonatal jaundice/hyperbilirubinemia, hypernatremia/dehydration, and hypoglycemia, all frequent causes of preventable hospitalizations for insufficient milk intake in breastfed neonates [4,5]. According to a large body of evidence, a sizeable minority of breastfed neonates receive inadequate nutrition/hydration [6-9]. Double-digit percentages of mother-infant dyads do not successfully establish breastfeeding within the first week and milk usually takes days to come in; intensive professional lactation support does not change those facts. The problem is particularly pronounced in first-time mothers, who some estimates suggest experience delayed onset of full milk production (lactogenesis II), with 33-44% of such mothers perceiving milk coming in beyond 72 hours postpartum [9,10]; in another study, Neifert et al. found that 15% of first-time mothers had persistent insufficient milk even after three weeks despite intensive professional lactation support [6].

First-time and more exhausted mothers tend to have more breastfeeding problems, and the risk of autism may be correspondingly greater in first-borns [11-14] and those likely to have more exhausted mothers, including in cases of delivery complications, prolonged labor, breech presentation neonates [13,15], and maternal lupus [16,17]. Additional maternal risk factors for breastfeeding insufficiencies include metabolic conditions such as overweight/obesity [18], gestational diabetes [19], polycystic ovarian syndrome (PCOS), breast tissue abnormalities from hypoplasia (a condition without consensus medical definition that is associated with PCOS and characterized by incomplete breast tissue differentiation) [20], cosmetic surgery [6], birth interventions including Caesarean section, and age. Studies similarly link most of these factors with offspring autism risk [21-25]. More direct links are uncertain due to limited research on breastfeeding problems, particularly with a maternal health emphasis and biological rather than psychosocial orientation. Overall, the available evidence suggests current early infant feeding guidelines incur substantial possible risk for affected neonates [26], as deprivation and frank starvation contribute to preventable jaundice, worsen its severity, and prolong it in the absence of close medical monitoring for near- and full-term neonates (as opposed to their preterm counterparts) - all of which may cause or contribute to brain injury resulting in a continuum of associated neurodevelopmental harms including autism spectrum disorder (ASD) [27,28].

Rising rates of autism [29,30] and other neurodevelopmental disorders [31] coincide with rising breastfeeding rates since the mid-1970s [32-36]. Historically, before its modern resurgence, the pendulum had swung away from breastfeeding in many modern, Western societies for generations. This created intergenerational knowledge gaps about safe breastfeeding. Well-meaning reformers then brought breastfeeding back with a new, historically anomalous emphasis on exclusivity, and in the absence of the pre-existing safety infrastructure - wetnursing [37-41], co-nursing [42], and prelacteal feeding [43-56] - that had protected neonates from common breastfeeding insufficiencies in all prior advanced civilizations. This safety infrastructure persisted in most of the non-Western world, including among the vast majority of foraging societies documented in the Human Relations Area Files (HRAF); in most societies in the HRAF and Standard Cross-Cultural Sample, mothers initiate breastfeeding at least a day and up to a week postpartum [57,58]. This contrasts with the modern, Western ideal of immediate postpartum initiation of birth mother breastfeeding. Where breastfeeding practices incorporating prelacteal feeding and shared nursing norms had been largely replaced by modern formula-feeding, the subsequent introduction of "exclusive breastfeeding" without an attendant safety infrastructure addressing common breastfeeding insufficiencies [26] led to an epidemic of common and preventable harm to neonates from insufficient nutrition/hydration [27,59-67].

The evidence on neonatal jaundice and autism suggests that rising autism rates may be part of that epidemic. As Amin et al. observe, if unconjugated hyperbilirubinemia is a significant cause of autism, it "is easily testable, implies prevention, and is of public health importance." The precautionary principle, acting to prevent harm when risk is uncertain and stakes are high, applies. Neonates should drink appropriate supplemental milk early, adequately, and often. This practice should be standard of care whenever insufficient breastmilk intake is suspected, particularly in cases of jaundiced neonates. This usually entails formula-feeding due to factors including limited banked donor breastmilk availability and infectious disease transmission concerns [26]. Minimizing neonatal deprivation and starvation periods should be standard in research and practice. A link between phototherapy treatment, the current standard treatment for neonatal jaundice, and possible autism risk further underscores the relevance of the precautionary principle, and the urgency of preventing and promptly treating the root cause of modal jaundice - insufficient milk intake in breastfed neonates - to prevent harm.

## Review

A PubMed search (1966 through February 4, 2022) for the terms "jaundice," "autism," and "meta-analysis" returned four published systematic reviews and meta-analyses. Of these, the first three comprehensively analyzed the relationship between jaundice and autism in the selected centrally indexed, peer-reviewed literature; this review details, synthesizes, and builds chiefly on their results, which consistently showed a substantial possible association between neonatal jaundice and autism. The fourth search result, a meta-analysis by Lai et al., focused on something different - the association between bilirubin and kernicterus spectrum disorder (KSD) - and limited its analysis of results relating to autism as a dependent variable to one study, which also showed a substantial possible association [68]. These articles, along with a few recent jaundice-autism studies not included in previous meta-analytical literature, suggest that jaundice may increase autism risk.

This review first summarizes the previous meta-analytical findings, which all show substantial possible risk. Second, this review arbitrates disagreement between these previous meta-analyses on the results of publication bias testing, noting common misuse of funnel plot tests for publication bias and presenting results of novel publication bias testing with a more modern tool, p-curve analysis. This analysis shows that results are unaffected by selective reporting (publication bias) and the underlying literature has evidential value. Hence, the jaundice-autism effect may be quite large and the link seems real; but where does the risk come from?

The link between jaundice severity and risk suggests that time matters; preventing jaundice progression may prevent harm. This is consistent with the evidence on global disparities relating to delayed healthcare access. The evidence on subgroup effects and global disparities further suggests that, in order to better understand where the risk comes from, we need to reconceptualize the dependent variable as a broader continuum of harm; existing estimates are likely underestimates, because other neurodevelopmental harms may occur alongside, or instead of, autism and patient-level mortality (survivorship bias) selects against severity. Other identifiable biases also likely bias existing estimates down: mild case inclusion dilutes the effect, study-level exclusion criteria select against severity, and study populations tend to over-represent wealthy, Western populations, under-representing populations likely to suffer disproportionate associated harm.

This body of evidence raises the question: If jaundice increases autism risk, time matters, and autism prevalence has substantially increased in recent history, then why might have untreated jaundice substantially increased in roughly the same period? The resurgence of breastfeeding from historical lows in modern societies, with novel emphasis on exclusivity and in the absence of previously widespread safety infrastructure guarding against common breastfeeding insufficiencies, offers a plausible explanation: neonates accidentally deprived of sufficient nutrition/hydration may suffer permanent harm [26-28].

Two puzzles in the jaundice-autism literature fit this story. First, meta-analyses report weaker associations in the preterm subgroup, a puzzle because preterms are more vulnerable. Different early infant feeding norms in preterms may make them the control group in the exclusive breastfeeding natural experiment. Second, phototherapy, the current standard treatment for neonatal jaundice, is associated with possible increased autism risk of greater magnitude than that associated with jaundice. The prior norm that phototherapy replaced was treating the root cause of modal neonatal jaundice, insufficient milk intake, through feeding neonates early, adequate, and often supplemental milk. Moreover, phototherapy appears to depend on excretion for its efficacy, and possibly safety, which insufficient milk intake compromises; many researchers have raised questions about possible risks associated with phototherapy, including increased autism risk. The evidence is consistent with the possibility of preventable neurodevelopmental harm from untreated insufficient milk intake in neonates treated with phototherapy for jaundice.

Jaundice-autism meta-analyses: consistent findings of substantial possible risk

Amin et al., Jenabi et al., and Kujabi et al. consistently found that jaundice in near and full-term neonates may risk substantial autism increase [1-3]. Their overlapping confidence intervals based on included studies’ pooled ORs estimated possible risk increases up to 67%, 68%, and 76%, respectively.

Amin et al. reported the results of a systematic review and meta-analysis on jaundice and autism including 13 studies retrieved from PubMed and MEDLINE databases published until 2009, mostly using retrospective matched case-control designs [1]. In a random-effects model, they estimated that jaundice, as assessed by total serum bilirubin (TSB), is associated with a substantial possible increased risk of autism. Their confidence intervals for the pooled meta-analysis of 13 examined studies (OR 1.43, 95% CI 1.22-1.67) showed a risk increase of 22-67%.

Jenabi et al. reported results of a systematic review and meta-analysis on jaundice and autism, updating and replicating the analysis by Amin et al. They included 21 studies, five cohort studies, and 16 case-control studies published up to April 2018 indexed in PubMed, Scopus, and Web of Science databases. They characterized their analysis's jaundice-autism correlation as "considerable" and, like Amin et al., observed that some studies reported the relationship was dose-responsive. Their confidence intervals for pooled odds ratio estimates (OR 1.35, 95% CI 1.02-1.68) showed a possible risk increase of 2-68%, with a larger possible effect size for the pooled estimated crude than adjusted OR (1.75 (.96-2.54) versus 1.19 (1.07-1.30)), and pooled risk ratio (RR) estimates (RR 1.39 (1.05-1.74)) showing jaundiced neonates are up to 74% more likely to develop autism [2].

Kujabi et al. reported results of a systematic review and meta-analysis on jaundice and autism replicating the core findings of Amin et al. and Jenabi et al. They examined 32 studies published up to February 2019 indexed in Pubmed, Scopus, Embase, Cochrane, and Google Scholar, limiting their analysis to six studies after deeming nine low risk-of-bias. This analysis reiterated that jaundice is associated with a substantial possible increase in later autism [3]. Of the six studies, analysis of the three identified low risk-of-bias cohort studies (RR 1.09, 95% CI .99-1.20) showed a risk decrease of -1% to an increase of 20%, and analysis of the three identified low risk-of-bias case-control studies (OR 1.29, 95% CI .95-1.76) showed a risk decrease of around -1% to increase of up to 76%. These values, coming from the highly restricted sampling of studies the authors considered best, suggest a sizeable possible autism risk increase. Kujabi et al., however, misinterpreted these results as showing "no association" and lack of "convincing evidence."

This set of meta-analytical results is consistent with possible underestimation (downward bias) in the literature. The most restricted meta-analysis of the three yielded the highest risk increase estimates, which might be because studies of lower quality generate more biased estimates. What do the included studies and underlying data demonstrate about the likely valence of bias in the literature? Previous reviews and meta-analyses have bracketed this question.

Amin et al. did not use a structured risk-of-bias or quality assessment to sort studies, a design choice consistent with relevant methodological recommendations. For instance, Greenland and O'Rourke cautioned that such quality scores have tended to poorly predict study results and are commonly misused in meta-analyses that fail to account for the underlying entities they measure (e.g., failing to account for the direction of the induced bias - virtually nullifying the value of bias/quality assessments), treat quality as low- or one-dimensional when it is many, and fail to weigh quality against formal bias-variance trade-off methods such as hierarchical (random-coefficient) meta-regression [69]. Low-dimensional appraisals, they emphasized, are unlikely to ever be adequate.

Building on Amin et al., Jenabi et al. followed the updated Assessing the Methodological Quality of Systematic Reviews (AMSTAR) guidelines [70] for reviews of non-randomized studies by assessing the impact of study-level risk-of-bias on the review results. They used the Newcastle Ottawa Scale (NOS) [71] to assess the quality of analyzed studies and categorized them as low or high quality. They did not consider the likely direction of bias.

Similarly, Kujabi et al. performed quality assessment including using the NOS. They reported being guided in this quality assessment by the Cochrane Handbook for systematic reviews of interventions [72] and the Strengthening the Reporting of Observational studies in Epidemiology (STROBE) checklist [73]. The relevant Cochrane Handbook chapter is Chapter 25: Assessing risk of bias in a non-randomized study [74]. It includes information on a bias assessment tool, Risk Of Bias In Non-randomised Studies - of Interventions (ROBIN-I). This tool "includes an optional component to judge the direction of the bias for each domain and overall," noting that while "for some domains, the bias is most easily thought of as being towards or away from the null... for other domains (in particular confounding, selection bias and forms of measurement bias such as differential misclassification), the bias needs to be thought of as an increase or decrease in the effect estimate to favor either the experimental intervention or comparator compared with the target trial, rather than towards or away from the null." The STROBE checklist similarly includes the item "Discuss both direction and magnitude of any potential bias" [73]. However, instead of discussing direction and magnitude of potential bias, Kujabi et al. noted their quality "assessment (aims) to show the quality of the studies without suggesting how that might influence the effect estimates." Bracketing the valence and substantive import of bias while relying on risk of bias as a binary quality metric leaves open questions about both bias and quality.

Thus, this present review fills a hole in the meta-analytical jaundice-autism literature by addressing previously bracketed questions about likely bias valence and practical significance. It identifies numerous likely underestimation biases: Mild case inclusion dilutes the effect, study-level exclusion criteria and patient-level mortality (survivorship bias) select against severity, other neurodevelopmental harms may occur alongside or instead of autism, and study populations tend to over-represent wealthy, Western populations, under-representing populations likely to suffer disproportionate associated harm. This matters because it suggests an even more substantial possible link between jaundice and preventable harm to neonates than the one consistently found by jaundice-autism meta-analyses, underscoring the sizeable possible magnitude of the effect and the importance of taking the link seriously. It should also be noted a priori that effect estimate bias could cut multiple ways (generating underestimates, overestimates, or both simultaneously along different or related pathways), and that "absence of evidence is not evidence of absence," in Altman's classic configuration [75]. Together, these facts mean that the scientific evidence on the jaundice-autism link, as usual in human affairs and risk estimates, contains uncertainty; this typical uncertainty about risk in a high-stakes context with sufficient evidence mandates preventive measures.

While these previous meta-analyses agreed neonatal jaundice may be associated with substantial autism risk, they disagreed on the interpretation of the results of publication bias tests. So the effect may be quite large, but is it real - or is there evidence that selective results reporting biased the literature?

Significant jaundice-autism findings demonstrate evidential value: results of publication bias testing with p-curve analysis

Meta-analyses often include publication bias tests, assessing the underlying literature for evidence of selective results reporting of significant versus null results (the "file-drawer problem") [76]. All three previous jaundice-autism meta-analyses used funnel plot tests for publication bias. All showed the same resulting pattern of two subgroup clusters, with a top center cluster reflecting lower estimates and a bottom right quadrant cluster reflecting substantially higher ones.

This is consistent with Sterne et al.’s warning that subgroup effects can hide in such plots [77]. Kujabi et al.'s shading suggest the top center cluster includes at least a few of the cohort studies while the bottom-right appears to be composed of case-control studies. This pattern is consistent with the dilution effect from inclusion of mild cases that Amin et al. observed [1]. It would make sense for cohort studies to be disproportionately affected, since population registry-type studies are likely to contain relatively complete universes of cases.

Despite displaying a markedly similar pattern of results, the first two meta-analyses reported divergent publication bias test results from the third. Amin et al. and Jenabi et al. reported no evidence of publication bias, while Kujabi et al. reported that the funnel plot test demonstrates high risk of publication bias in all studies. Kujabi et al. misinterpreted the test results; all three meta-analyses misused the test and none fully reported related statistical test results, which are essential for interpretation.

A best possible use-case for this test would be with randomized controlled trials performed under similar conditions in similar settings, so that the distribution of variance could be expected to be symmetrical but for selective results reporting; by contrast, the jaundice-autism literature presents a use-case closer to the worst possible end of the spectrum, composed of observational studies defining key constructs differently and heterogeneity accordingly high. Thus, funnel plot tests for publication bias are inappropriate in this context, because the correct statistical conditions for using them are absent. The test's fundamental assumption of symmetrical distribution of variance, as in a hypothetical sample of randomized controlled trials, could reasonably be expected to be violated in this observational context of studies with high heterogeneity; but such asymmetry from heterogeneity, as in the case of the established dilution effect likely contributing to the observed subgroup clustering, would not demonstrate risk of publication bias. In addition, the plots all showed visual asymmetry, which does not necessarily indicate statistical asymmetry; formal statistical tests are needed to distinguish the chance appearance of asymmetry from underlying asymmetry [78]. Amin et al. and Jenabi et al. reported performing such tests; Kujabi et al. did not. Finally, neither visual nor statistical funnel plot asymmetry accurately predicts publication bias [79].

Such misuse of funnel plot tests for publication bias is so widespread as to be normal in the medical literature. In a large survey evaluating all Cochrane Database of Systematic Reviews meta-analyses of binary outcomes with three or more studies, Ioannidis and Trikalinos find that most medical journal meta-analyses that use publication bias tests make this mistake: "Statistical conditions for employing asymmetry tests for publication bias are absent from most meta-analyses; yet, in medical journals, these tests are performed often and interpreted erroneously" [80]. This review replicates and extends that result to the jaundice-autism meta-analytical literature.

This problem illustrates and is part of the larger crisis in science, with perverse incentives undermining the knowledge system that is supposed to underpin medicine and public policy [81-84] and prominent calls to change the incentive structure ongoing for decades [85,86]. The persistence of common mistakes, such as misuse of statistical tests including those for significance and publication bias, illustrates both the ongoing nature of the crisis and how easy amelioration might be; retiring statistical significance thresholding and banning misuse of funnel plot tests for publication bias seem like simple reforms for methods experts to recommend and journals to implement.

Ironically, such publication bias tests are intended to address one particular facet of the crisis in science, the reproducibility crisis [87], in which most important results in scientific fields ranging from biotechnology, hematology, and oncology to psychology cannot be replicated. In this context, Bishop suggests publication bias is only one of the "four horsemen" of irreproducibility, with low statistical power, p-value hacking, and HARKing (hypothesizing after results are known) also threatening the integrity of science [88]. This suggests two insights: first, addressing the crisis in science with ever more quantitative rules and checklists seems to sometimes backfire, undermining the integrity of the literature it was intended to promote; observers have long argued it is the underlying incentive structure that must change, and the failure of technical fixes to promote quality science supports that position. And second, while the funnel plot test for publication bias should not be applied to the jaundice-autism literature because the correct statistical conditions for employing it are absent, the other horsemen of irreproducibility could be considered.

The current review considers low statistical power (underpowering) and p-value hacking. But it should also be noted that there may be other, entirely benign reasons for different studies in the underlying jaundice-autism literature producing different effect sizes. For instance, different ways of defining key constructs could generate substantial heterogeneity, as in the mild case inclusion dilution effect. In addition, possible confounding presents challenges to inferring causality but does not threaten the validity of correlational effect estimates. Insufficient milk intake, the modal root cause of neonatal jaundice, also causes hypoglycemia and hypernatremic dehydration, both of which can cause permanent neurological damage; an apparent jaundice-autism link could result in part from these common comorbidities. But different hospital, country-level, or other norms could generate substantial variation in monitoring for and treatment of these conditions, generating outcome differences. In both examples, selective results reporting is not needed to explain the fact that different studies generate different effect estimates.

P-curve analysis, a meta-analytical test for evidence of p-value hacking, presents a more modern technique in publication bias testing with relevance to the jaundice-autism literature. It detects selective results reporting by making inferences from the shape of "the distribution of statistically significant p-values for a set of independent findings" [89]. This is important because it lets us know whether a set of significant findings "contains evidential value when we can rule out selective reporting as the sole explanation of those findings." Left-skewed p-curves suggest substantial p-hacking (manipulation of significance testing to find positive results), p-curves that are not skewed suggest lack of evidential value of findings, and right-skewed p-curves "are diagnostic of evidential value" [90]. P-curve testing has numerous limitations, including ignoring nonsignificant results and thus likely producing noisy effect estimates, although this limitation does not make p-curve biased [91], and the method can also bias effect estimates down. These and other limitations [92] suggest p-curve is not an appropriate tool to estimate average effect size. Average effect size estimates should not be interpreted literally in this context anyway, since factors like jaundice severity need to be accounted for, and - a common caveat in scientific evidence reviews relevant to public policy - methodological limitations of available data do not support literal interpretation of point estimates. The appropriate use of this tool in this context is to look at the distribution of p-values, and see if it seems to substantiate or trouble the evidential value of the analyzed literature with respect to potential publication bias, since "p-curve fully corrects for the impact of selectively reporting studies" [91]. So what does the p-curve of jaundice-autism studies show?

This analysis applies Simmons et al.’s study selection guidelines [89], considering for inclusion all PubMed-indexed studies (as of January 3, 2022) that meet published jaundice-autism meta-analysis inclusion criteria (numbering 36). The current review considers more recent studies by Lee et al. [93] and Cordero et al. [94] that previous meta-analyses did not consider, excluding Cordero et al since it does not report p-values. Several studies were excluded because their p-values were statistically non-significant, and p-curve is "the distribution of statistically significant p-values for a set of studies" [89]. Nath et al. is excluded for full-text article inaccessibility [95]. Studies by Sugie et al. [96], Maimburg et al. [97], Finegan-Quarrington [98], Juul-Dam et al. [99], Chien et al., and El baz et al. were excluded for not reporting specific p-values. Lord et al. was excluded due to a lack of jaundice-specific results reporting [100]. This left 10 studies for inclusion in the p-curve analysis (Table 1): Lee et al. [93], Chen et al. [101], Maimburg et al. [102], Bhattarai et al. [103], Lozada et al. [104], Duan et al. [105], Froehlich-Santino et al. [106], Mamidala et al. [107], Zhang et al. [108], and Ahmed et al. [109]. This analysis follows the Official User-Guide to the P-curve [110], including for structuring the P-curve Disclosure Table (Table 1); it generates and reports results using the p-curve app version 4.06 [111].

**Table 1:** Studies Included in p-Curve Analysis IUGR: intrauterine growth retardation; CFA: craniofacial anomalies; aOR: adjusted odds ratio; HR: Hazard Ratio; ASD: autism spectrum disorder; ADHD: attention deficit hyperactivity disorder

Original papers	Quoted text from original paper indicating prediction of interest to researchers	Study design	Key statistical result	Quoted text from original paper with statistical results	Results
Lee et al., 2021 [93]	In the present study, we investigated insidious etiology factors during the perinatal period associated with autism.	Population-based case-control	Logistic regression	After logistic regressive analysis, the adjusted odds ratios of IUGR, CFA, neonatal hypoglycemia, and neonatal jaundice were 8.58, 7.37, 3.83, and 1.32, respectively.	Neonatal jaundice aOR 1.32 (95% CI 1.00–1.74), p = .021
Chen et al., 2014 [101]	To clarify the independent relationship between neonatal jaundice and developmental disorders, multivariate Cox regression analysis was performed to investigate the HR with 95% CI of ASD, ADHD, and other developmental disorders after adjusting gender, level of urbanization, and comorbid perinatal conditions.	Taiwanese population-based (insurance database) national cohort	Multivariate Cox regression analysis	Newborns with neonatal jaundice had increased risks of developing ASD (HR: 1.75, 95% CI: 1.05-2.90), any developmental delay (HR: 1.27, 95% CI: 1.02-1.58), and developmental speech or language disorder (HR: 1.41, 95%CI: 1.11-1.79).	ASD HR 1.75 (95% CI 1.05-2.90), p = .031
Maimburg et al., 2010 [102]	The goals were to study the association between neonatal jaundice and disorders of psychological development in a national, population-based cohort and to study whether gestational age, parity, and season of birth influenced that association.	Danish population-based (birth register) national cohort	Cox regression models with right censoring	The excess risk of developing a disorder in the spectrum of psychological development disorders after exposure to jaundice as a neonate was between 56% (HR: 1.56 (95%CI: 1.05-2.30)) and 88% (HR: 1.88 (95%CI: 1.17-3.02)). The excess risk of infantile autism was 67% (HR: 1.67 (95% CI: 1.03–2.71)).	Excess risk of infantile autism HR 1.67 (95%CI 1.03-2.71), p = .038
Bhattarai et al., 2018 [103]	To determine the demographic profile of patients diagnosed with ASD, determine the significant prenatal and perinatal risk factors associated with ASD.	Case-control	Univariate analysis, Chi-square test	The results showed that there was a significant association noted between neonatal jaundice (p=0.01) with ASD.	Seven cases (12.1%), zero controls, p = .01
Lozada et al., 2015 [104]	Utilizing a large US health care database representing a heterogeneous, demographically and socioeconomically diverse population, we sought to expand on previous smaller studies evaluating the risk of ASD among infants with a history of neonatal unconjugated hyperbilirubinemia (jaundice), while utilizing a more refined definition of both neonatal jaundice and ASD.	Case-control	Multivariate conditional logistic regression	After adjustment, there remained an association between ASD in children and an admission with a diagnosis of jaundice (OR = 1.18; 95%CI = 1.06-1.31; p = .001) and phototherapy treatment (OR = 1.33; 95%CI = 1.04-1.69; p = .008).	Excess risk of ASD in children admitted with a diagnosis of jaundice, OR 1.18 (95%CI 1.06-1.31), p = .001
Duan et al. 2014 [105]	The current study compared the perinatal and clinical data of 286 children with confirmed autism to similar data from 286 healthy age-matched children in central China. We then used single factor and multiple logistic regression to identify factors associated with autism risk.	Chinese case-control	Multivariate logistic regression	This analysis identified parental age, paternal introversion, passive smoking, premature rupture of the fetal membrane, premature delivery, birth asphyxia, and severe neonatal jaundice as independent risk factors (P < 0.05) for childhood autism in Han Chinese children of central China (Zhengzhou City).	Severe jaundice β value 3.201, OR 21.811 (95%CI 12.221-35.539), p = 0.009
Froehlich-Santino et al., 2014 [106]	We also explored whether individual perinatal factors were associated with the presence of ASDs in both concordant and discordant twin pairs. Amongst these, we believed low birth weight and markers of hypoxia would be associated with higher risks for ASDs.	Twin cohort	Logistic regression	In contrast, respiratory distress, jaundice, having an oxygen requirement after birth, and the presence of a marker for hypoxia were all significantly more common in twin individuals with ASDs than twin individuals without ASDs.	Jaundice OR 1.69 (95%CI = 1.09-2.62), p = 0.02, Bonferroni adjusted p = 0.13
Mamidala et al., 2013 [107]	A questionnaire was designed based on the probable risk factors of ASD from existing literature and those likely to be specific to the Indian environment. Care was taken to analyze and include the most relevant and specific risk factors and not a meager dependence on the already available data from literature.	Indian population-based retrospective cohort	Multivariable logistic regression	The factors that were significant after adjusted analysis were mother’s age at gestation (aOR: 1.59), fetal distress (aOR: 5.13), respiratory infections (aOR: 3.80), labor complications (aOR: 4.52), pre-term birth (aOR: 1.78), birth asphyxia (aOR: 10.63), neonatal jaundice (aOR: 2.89), and delayed birth cry (aOR: 2.68).	Jaundice OR 2.89 (95 CI 1.58-5.28), p = .0006
Zhang et al., 2010 [108]	To further investigate the relationship between autism and prenatal and perinatal conditions, and to identify factors potentially relevant to the etiology, and, ultimately, the prevention of this disorder, we conducted a case-control study using children with and without autism in China.	Chinese case-control	Simple logistic regression	Neonatal complications including apnoea and jaundice were extremely rare in our control group but much more common in our case group, consistent with the findings of Maimburg et al. that an almost fourfold increased risk for infantile autism was observed in newborns exposed to hyperbilirubinemia. (Maimburg et al. [97]).	Jaundice unadjusted OR 12.31 (95%CI 1.56-97.36), p = .005
Ahmed et al., 2010 [109]	The study aimed to investigate the relationship between neonatal hyperbilirubinemia and autism.	Saudi Arabian case-control	Chi-square test	All children with autism had jaundice after birth compared to less than half of the children in the control group. The difference was highly statistically significant (100% Vs. 42%, respectively, p ≤ 0.0001).	Chi-square test comparing jaundice in autistic and non-autistic group = 38.08, p = 0.0001

**Figure 1:**
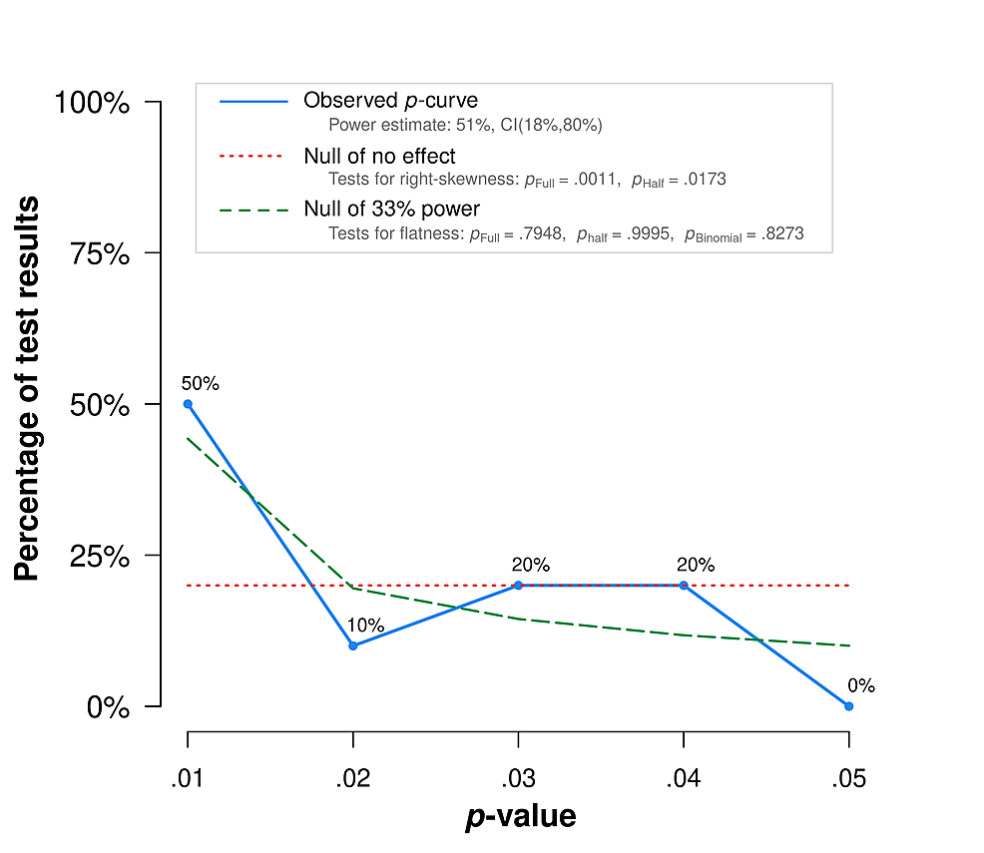
p-curve of Jaundice-Autism Studies The observed p-curve includes 10 statistically significant (p < .05) results, of which eight are p < .025. There were no non-significant results entered.

The p-curve exhibits right-skewness (Figure 1, Table 2). In particular, it meets the combination test that Simonsohn et al. [112] introduced: both the half and full p-curve tests are right-skewed with p<.1. This result suggests the included studies contain evidential value. There is no available evidence that studies linking jaundice and autism demonstrate publication bias. The link warrants further research and action. 

**Table 2:** Jaundice-Autism p-curve Analysis Results

	Binomial Test (Share of results p<0.025)	Continuous Test (Aggregate with Stouffer Method)	
		Full p-curve (p's<0.05)	Half p-curve (p's<0.025)
Studies contain evidential value. (Right skew)	p=.0547	Z=-3.05, p=.0011	Z=-2.11, p=.0173
Studies’ evidential value, if any, is inadequate. (Flatter than 33% power)	p=.8273	Z=.82, p=.7948	Z=3.32, p=.9995
Power of tests included in p-curve (correcting for selective reporting)	Statistical Power Estimate: 51%		
	90%CI: 18%, 80%		

So the jaundice-autism effect may be quite large and the statistically significant correlation seems real, but where does the risk come from? The evidence suggests that severity matters; however, there is no safe level of hyperbilirubinemia with respect to neurodevelopmental harm. Both findings indicate that the precautionary principle is particularly relevant to preventing associated harm.

Jaundice severity and autism risk: the case for preventing progression

Time matters; jaundice may already have caused irreversible neurological damage by the time it is identified, particularly when it has progressed. Mandic-Maravic et al. argue damage is done by the time of diagnosis [113]. Bhutani et al. note "once the early stages of hyperbilirubinemic brain damage occur, therapeutic options are limited to the prompt (<8h) use of exchange transfusion, hence the imperative for preventive approaches" in relation to severe neonatal jaundice [114]. Leaving jaundice untreated risks worsening severity, underscoring the need for prompt treatment (or better, prevention) to minimize harm.

Overall, the evidence is consistent with a possible dose-responsive jaundice-autism link wherein greater jaundice severity over a certain threshold poses greater neurodevelopmental risk. In recent reviews and meta-analyses on the jaundice-autism link, Amin et al. and Jenabi et al. noted evidence of a relationship between increased jaundice severity and possible increased autism risk; earlier reviews also presented findings that underscore the importance of preventing jaundice progression in order to prevent possible neurodevelopmental harm. Ip et al. observed that most cases of kernicterus (severe bilirubin-induced brain injury) were associated with bilirubin over 20 mg/dL [115]. Trikalinos et al. found TSB measurement shows reasonable predictive power, but screening does not necessarily improve clinical outcomes [116]; this is consistent with the possibility that damage is done by the time of diagnosis. In a recent systematic review and meta-analysis on the association between bilirubin and KSD, Lai et al. found TSB >25 mg/dL appears to increase the risk of KSD [68]. They conclude in agreement with Ip et al. [117] that other risk factors should be considered in combination with early TSB, consistent with the consensus view that there is no established safe level of elevated bilirubin in part due to subgroup variation in associated risks.

All three previous jaundice-autism meta-analyses (Amin et al., Jenabi et al., and Kujabi et al.) noted high heterogeneity. Amin et al. presented particularly insightful observations on subgroup effects with respect to severity. First, they noted "all five studies that demonstrated significant positive association... defined jaundice based on the magnitude of TSB concentration and evaluated association between ASD and moderate to severe jaundice (TSB > 10 mg/dl). In comparison, the majority of studies that demonstrated no association (OR<1) between jaundice and ASD included infants with any degree of jaundice" [1]. This suggests that worse hyperbilirubinemia correlates with more possible increased autism risk, over a certain severity threshold. Including mild jaundice cases in analyses appears to dilute the effect. Unless jaundice is treated as a non-binary variable or stratified into multiple binary dependent variables according to severity, this dilution from mild case inclusion is likely to bias jaundice-autism effect estimates down, particularly those based on datasets that may appear to be higher-quality because they include a more complete universe of jaundice data, something that Kujabi et al. does not appear to have considered. Second, Amin et al. noted, "most studies that demonstrated significant positive association between jaundice and ASD... were larger and more recent compared to studies that failed to demonstrate a significant association between jaundice and ASD." This suggests that studies with larger sample sizes and thus less vulnerability to Type-II errors from underpowering tend to report more significant effects. These observations are consistent with the possibility that studies demonstrating no jaundice-autism association (or no significant association, often misinterpreted as no association) may be false negatives.

These observations on heterogeneity are consistent with a dose-responsive jaundice-autism link, which is consistent with a causal role of jaundice in autism development via brain injury resulting in bilirubin-induced neurological dysfunction (BIND). BIND is a continuum that may also include attention deficit hyperactivity disorder (ADHD) [118,119], autism, cerebral palsy [120-122], cognitive and developmental delay and disorder [101], epilepsy [123,124], hearing impairment [125,126], kernicterus [27], language disorder, mood disorders [127], and lower IQ and specific learning disorder [28]. Recent trends toward entertaining causal links build on a tradition including Simon's suggestion that autism might be a variant of kernicterus [128] and Shapiro's observation that, as more became known about the neurobiology of kernicterus, definitions evolved to include athetoid cerebral palsy, impaired upward gaze, deafness, auditory neuropathy or dys-synchrony, and subtle BIND, with some suggesting moderate hyperbilirubinemia [129] may increase risk of later ADHD, autism, and Parkinson's disease.

In later research, Amin et al. reviewed the observational evidence linking the BIND spectrum and some comorbid neurodevelopmental disorders, delineating multiple possible biological mechanisms linking jaundice with harm [28]. As they noted, the relationship between neonatal jaundice and neurodevelopmental risk might be better estimated by a broader conceptualization of potentially associated harms to include a continuum rather than one, relatively narrowly operationalized binary outcome. Estimates focusing exclusively on autism are thus likely underpowered to measure the true outcome of interest, preventable harm associated with neonatal jaundice.

Dose-responsive relationships are often non-linear, with risk rising rapidly over a certain exposure threshold, and rare but serious risks affecting a small minority of individuals. Although evidence suggests jaundice severity tends to correlate with increased risk of neurodevelopmental harm, the low base rate of kernicterus, as well as outlying cases of permanent damage from relatively low levels of hyperbilirubinemia, trouble the notion of a clinically relevant linear relationship between bilirubin level and brain injury. The bottom line clinically is that there is no established safe level of neonatal jaundice.

The evidence supports applying the precautionary principle by feeding hungry neonates formula to prevent and treat jaundice and other common complications of insufficient milk intake, which may risk neurodevelopmental harm [26]. Supplementation with water or sugar water does not reduce hyperbilirubinemia [130,131]. Formula lowers bilirubin by inhibiting intestinal reabsorption [132]. Formula-feeding in places with clean water, high literacy, and stable formula access is well established as safe and is likely often also a safer option than prolonged neonatal starvation in lower-resourced settings where medical care access to treat complications of insufficient milk intake tends to be more limited. By contrast, jaundice treatment options such as phototherapy and exchange transfusions are invasive, costly, potentially stressful for neonates and their families, and may incur substantial risk. In infants who are already medically endangered by moderate to severe jaundice, these interventions may be necessary to limit brain injury and disability; but it is uncertain to what extent damage is already done by the time of diagnosis. In any event, these cases are overwhelmingly preventable through supplementing breastfeeding with formula prophylactically, or in response to signs of persistent infant hunger at the latest. Formula-feeding appears safer for neonates than common complications from insufficient milk intake and current standard treatments for them.

Neonatal jaundice severity is risky, and supplementation prevents modal jaundice from progressing by treating its root cause of insufficient milk intake. So does a little bit of supplementation eliminate the risk? Evidence shows that exclusive breastfeeding is the riskiest infant feeding practice with respect to hospitalization for complications of insufficient milk intake [65-67]. Even though some supplementation is better than none when it comes to avoiding the worst outcomes of neonatal starvation, insufficient supplementation remains risky. Shan et al. report rooming-in is associated with increased exclusive breastfeeding, birth weight loss >10% at day three of age, and neonatal admission for phototherapy for hyperbilirubinemia [67]. Both exclusively breastfed and mixed-fed neonates alike in this sample ran increased hospitalization risk under a policy regime designed to promote exclusive breastfeeding in line with Baby Friendly, the WHO/UNICEF program of exclusive breastfeeding promotion. This suggests even supplemented neonates may frequently suffer medically inadequate nutrition/hydration under Baby Friendly-style policies, which educate mothers on benefits but not risks of exclusive breastfeeding and discourage supplementation unless "medically necessary" [135]. 

There is insufficient evidence to establish which neonates medically require formula and how much is then medically necessary. This is illustrated by preventable hospitalizations for hyperbilirubinemia in Flaherman et al.’s randomized controlled trials limiting or denying early formula supplementation to neonates with excessive weight loss defined as weight loss ≥5% but <10% of their birthweight at 24-48 hours old [136] or being in the ≥75th percentile for weight loss at age at 24-72 hours [137]; denial appears to cause more harm than supplementation, but sometimes the amount of supplement offered is also medically insufficient and hospitalization results. By prioritizing the restriction of formula over ensuring neonates drink sufficient milk, the exclusive breastfeeding paradigm is associated with increased risks for mixed-fed as well as exclusively breastfed neonates. Supplementation should be sufficient to prevent harm, and the evidence establishes neither that neonates lack a homeostatic capacity to regulate milk intake, nor that colostrum or a particular amount of supplemental milk is sufficient. “Sufficiency" lacks a consensus medical definition with respect to either parameters of common relevant complications such as jaundice, hypoglycemia, and hypernatremic dehydration, or later neurodevelopmental harm possibly associated with such complications in the neonatal context, such as autism, epilepsy, and hearing impairment.

So severity matters and jaundice can progress over time, even with limited supplementation. That means time matters; but then is it jaundice severity or duration that drives risk? While we have theoretical and empirical reasons to suspect that moderate to severe jaundice is riskier for neonates than mild jaundice in neurodevelopmental terms, similar analyses do not appear to have been performed for jaundice duration. On one hand, the two can be closely related. Jaundice duration can worsen severity, e.g., when insufficient milk intake is a persistent contributing cause. On the other hand, the open empirical question of whether jaundice duration independently contributes to neurodevelopmental risk seems especially important, since healthcare practitioners routinely advise new mothers to continue exclusively breastfeeding jaundiced neonates.

Minimal research addresses this question. Ahmed et al. observed in a Saudi Arabian case-control study that mean days of jaundice predicts autism, and the difference is large in mean days (19.6 ± 5.4 for the autism group versus 4.08 ± 7.115 for the control group) and highly statistically significant (p = 0.000) [109]; but this analysis neither disambiguates duration and severity, nor addresses etiology. Further research could attempt to disambiguate between jaundice duration and severity in increasing later risk of autism and other associated harm. Etiology also warrants further study in this context. Are duration and severity equally risky across subgroups with jaundice of different likely origins, such as neonates with infections versus those not receiving insufficient nutrition/hydration due to breastfeeding insufficiencies?

The answers to such questions may be academic insofar as the evidence is already sufficient to invoke the precautionary principle: early, adequate, and often supplementation, combined with increased monitoring and continued breastfeeding support when desired, should replace the exclusive breastfeeding paradigm to prevent common harm to neonates. This change in early infant feeding recommendations may paradoxically increase breastfeeding in some subgroups. The reason is that insufficient milk intake weakens neonates, just as lack of adequate nutrition and hydration weakens adults and other animals. So neonates starved for days waiting for their mothers' milk to come in may not be strong enough to drink sufficient milk when mature milk production starts. Muscle weakness and lethargy from starvation could cause weaker sucking, signaling mothers to produce less milk. Exclusive breastfeeding promotion may thus contribute to a vicious cycle undermining breastfeeding success, particularly for neonates who are more sensitive to the effects of starvation (e.g., due to genetic propensities to jaundice or hypoglycemia) and mothers whose milk takes longer to come in (e.g., primaparas).

The apparent relevance of jaundice severity level to neurodevelopmental risk highlights a source of possible underestimation bias in the jaundice-autism literature. Many studies use exclusion criteria that will tend to exclude more severe jaundice cases [95-6,105,107]. Those criteria include Rh factor isoimmunization or blood diseases - both risk factors for severe jaundice, as well as visual or hearing impairments, cerebral palsy, and delay in motor developments - all potential components of BIND (i.e., jaundice-associated neurodevelopmental harm).

Jaundice severity and global disparities: subgroup effects and unrepresentative samples

The relationship between jaundice severity and harm suggests additional ways in which the existing literature may tend to underestimate associated deaths and disabilities. The continuum of jaundice-associated neurological harm that Amin et al. proposed should be further extended to include the jaundice-associated deaths that are substantially more common in poorer countries. Over-representation of well-resourced countries in the literature, together with over-narrow operationalization of the outcome of interest stemming from that perspective, likely introduces selection bias substantially driving down estimates of associated harm.

Severe jaundice-associated harms disproportionately affect many subgroups along some usual lines of structural power disparity, with racial and geographic outcome differences overlapping socio-economic and international developmental ones; greater male vulnerability is the obvious exception. Kernicterus disproportionately affects Black babies [138]. Autism disproportionately affects vulnerable groups including Black and non-English speaking pupils in England [139] and African-Americans in the United States (US) [140]. In a narrative review, Tromans et al. [141] found that "minority ethnic groups" tend to be diagnosed with more severe autism, but diagnosis appears less frequent in these groups, suggesting that healthcare access, environmental influences, and cultural factors may play a role in under-diagnosis of higher-functioning minority children with autism.

Racial subgroups differences, and misconceptions about them, likely play a role in perpetuating such disparities by causing diagnostic failures. In a previous review, Wilde [26] found a lower threshold of concern is appropriate for jaundice in the context of prematurity [142], low birth weight, systemic infection such as sepsis, birth trauma [143], C-section, male gender [144], Asian race due to increased incidence of *UGT1A1* or *SLCOs* polymorphisms [145-147], African, Sephardic Jewish, Greek, Turkish, Chinese, and Italian races due to increased risk of glucose-6-phosphate dehydrogenase (G-6-PD) deficiency [148], and darker skin due to melanin reducing visible skin yellowing [149]. While a lower threshold of concern is thus appropriate for African/Black, Asian, and other darker-skinned infants, in practice, a higher threshold of concern may instead sometimes still prevail, because it used to be widely believed that Black babies were at lower risk for jaundice and associated harms [148,150]. In addition, many of these heightened-risk categories tend to overlap (e.g., preterm and Black) [151], and we do not know how they interact in terms of potentially magnifying risks of harm associated with neonatal jaundice.

Disparities in healthcare access also contribute to outcome disparities. Infants from low- and middle-income countries more frequently suffer from severe hyperbilirubinemia [152] due in part to later diagnosis, treatment delays, and lower-quality care including excessive use of belated (ineffective and risky) exchange transfusions [153]. In a review, Cayabyab and Ramanathan found that exclusive breastfeeding is an under-recognized risk factor for severe neonatal jaundice [152]. Slusher found that lower-resourced countries are disproportionately affected by permanent disability and death associated with severe hyperbilirubinemia, estimating Africa has the highest severe neonatal jaundice incidence at 668/10,000 live births versus the American and European incidence of 3.7/10,000 [143]. In Nigeria, acute bilirubin encephalopathy (ABE), an acute illness caused by severe hyperbilirubinemia, causes 5-14% of neonatal deaths [154-156]. A study of nine major hospitals in five cities found over 15% of infants treated for hyperbilirubinemia had mild to severe bilirubin encephalopathy (including 35 deaths) [157]. Increased exclusive breastfeeding and attendant lower prelacteal feeding are associated with increased neonatal mortality in Burkina Faso [158-160], but available data do not identify causes of death.

Thus, survivorship bias may cause substantial jaundice-autism effect underestimation in lower-resourced settings. In their later review, as previously noted, Amin et al. recognized that underpowering of jaundice-harm estimates is likely due to overly narrow focus on autism to the exclusion of potentially related neurodevelopmental risks [28]. They favored reconceptualizing a continuum of harms on the BIND spectrum. This continuum should include jaundice-associated deaths. These deaths are likely to affect poorer countries, preterms, and especially preterms in poorer countries in a vastly disproportionate manner. For example, Nabwera et al. reported high mortality amongst preterms in Kenya and Nigeria [161], countries where the burden of severe neonatal jaundice is also high.

Later access to less appropriate care means that the safety advantages of supplemental feeding relative to starvation are likely more marked for neonates in lower-resourced settings compared to their wealthy, Western counterparts. This may help explain why widespread prelacteal feeding, seen by some of its practitioners as helping new mothers rest and newborns stay nourished to promote health and breastfeeding success, has a long history of diverse traditions and continues today, along with more limited wetnursing and co-nursing practices, in many low- and middle-income countries [43-56] and most foraging societies [58]. Such supplementary feeding is the target of extensive public health attention aiming to stop it. But evidence suggests it may be safer for neonates than exclusive breastfeeding in the absence of safety infrastructure protecting them from common breastfeeding insufficiencies associated with clinically significant harms, particularly in settings where lack of access to timely and appropriate medical treatment for related complications is more likely to result in death or permanent disability.

This split between contemporary early infant feeding practices in the modern West and elsewhere may help explain why earlier observers like Sanua [162] thought autism was a modern Western disease. Sanua suggested, "infantile autism appears to be an illness of Western civilization and appears in countries of high technology, where the nuclear family dominates." Autism caused by jaundice associated with exclusive breastfeeding may well have been a modern Western disease in this sense, at least before public health initiatives succeeded in more aggressively spreading the new early infant feeding paradigm worldwide.

At the same time, the implied contrast point - non-modern Western settings - is heterogeneous, especially in an increasingly globalized world, and this matters for likely bias valence and magnitude in overall jaundice-autism estimates in terms of the literature's unrepresentativeness of the global population. In some settings, such as most foraging societies, neonatal jaundice prevention may be so routine through cooperative breastfeeding practices that associated neurodevelopmental harm does not occur. These settings contain a very small minority of the world's population, with Burger and Fristoe estimating the global hunter-gatherer population at around 10 million [163]. Were this population far more numerous, this might conceivably drive overestimation bias in the jaundice-autism literature, with non-Western settings with traditional early infant feeding practices relatively protected from jaundice and its harms (ironically in light of lack of modern medical care access). But a far greater proportion of the world's population lives in non-modern Western contexts that are influenced by modern Western norms and other forces in myriad ways; the population of sub-Saharan Africa alone exceeds one billion [164]. These majority non-Western, educated, industrialized, rich, and democratic (non-WEIRD) [165] contexts, while still home to a range of common prelacteal feeding practices, tend to have been targeted by successful public health interventions promoting exclusive breastfeeding, as in the Promoting Infant Health and Nutrition in Sub-Saharan Africa: Safety and Efficacy of Exclusive Breastfeeding Promotion in the Era of HIV (PROMISE-EBF) trial [158-9], conducted from 2006-2011 in Burkina Faso, Uganda, South Africa, and Zambia. In such settings, the international community's introduction of modern Western early infant feeding norms may combine with various forms of resource poverty, including lack of healthcare access, to substantially magnify risks of serious jaundice-associated harm, as possibly reflected by the highest-ever measured perinatal mortality rate in Burkina Faso [158].

In sum, better-resourced settings are over-represented in the literature. That unrepresentativeness affects all jaundice-autism meta-analyses, compounding likely underestimation of associated harm. All jaundice-autism meta-analysis authors recognized that possible confounding and selection bias associated with observational studies plague meta-analyses like theirs. But, at the same time, the authors all bracketed bias valence, so this probable underestimation tendency does not appear to have been previously identified.

That said, Jenabi et al. and Kujabi et al. compiled summary results of included studies that show that they took place in countries that would be vastly disproportionately coded WEIRD, Organisation for Economic Co-operation and Development (OECD), more developed, and/or higher-income. Sub-Saharan Africa and South America are entirely absent, most of the rest of Africa and Asia are vastly underrepresented, and Scandinavia is over-represented within the European region. This distribution suggests that relevant systematic biases in healthcare access are highly likely and consequential. Olusanya et al. estimate that the largest number of neonatal deaths worldwide are frequently accounted for by Sub-Saharan Africa and South, East, or Southeast Asia, with bilirubin-induced mortality common [166]. Thus, this selection bias is practically significant for studies of neonatal jaundice-associated harm. Most related infant deaths are invisible; these deaths are largely uncountable due to a lack of data, and they are numerous.

Selection bias in favor of better-resourced settings thus seems likely to limit included studies' and related meta-analyses' generalizability to similarly-resourced settings. Since we can guess the likely direction of bias (downward) and other practical implications (underestimation of practically significant effects including substantial preventable infant morbidity and mortality), this highlights the importance of taking seriously findings that estimate substantial possible harm from jaundice. They likely underestimate harms, and this underestimation likely disproportionately impacts vulnerable groups.

These harms vary not only by race and place as described above but also by historical time. As Amin et al. concluded, "unconjugated hyperbilirubinemia may be linked to ASD and may have affected the [rising] prevalence of ASD globally" [1]. This would imply a rise in neonatal jaundice, which the rise in "exclusive breastfeeding" could explain. Lack of data on breastfeeding problems and neonatal starvation periods makes direct tests impossible, but what does the available evidence suggest about this historical coincidence?

Rising autism prevalence: causes, estimates, and historical coincidence with the exclusive breastfeeding paradigm

Exclusive breastfeeding along the starvation jaundice pathway is only one of many possible contributors to rising autism rates. Future research should undertake a full exposition of all these factors with preventive emphasis, integrating available evidence on early insufficient milk intake and neurodevelopmental risk into available evidence on perinatal risk factors and autism. Reviewing the latter, Cheng et al. state that prolonged breastfeeding is reportedly linked to possible autism risk reduction [167]. The authors do not appear to consider the possibility that this finding could be due to women for whom breastfeeding works earlier and better opting to breastfeed longer than their counterparts with breastfeeding insufficiencies. But we know that delayed onset of lactogenesis II predicts excessive neonatal weight loss, formula supplementation, and earlier breastfeeding cessation [6,10], and insufficient milk has long been among the most commonly cited reasons for early weaning worldwide [57,168-171]. Thus data on autism risk appear consistent with the hypothesis that breastfeeding problems can cause insufficient nutrition/hydration, and exposure to such conditions of deprivation/starvation can harm neurodevelopment.

Other possible contributors to rising autism incidence also warrant full exploration with a harm prevention focus. Some relate to the breastfeeding starvation hypothesis directly (as in other complications of insufficient milk intake), others indirectly (as in other facets of the modern Western condition). Chief among the directly related possible contributors are hypoglycemia and hypernatremia, which can also cause brain damage and are also often associated in previously healthy near- and full-term neonates with insufficient milk intake, although jaundice appears to be the complication most commonly diagnosed. Chief among the indirectly related ones are greater cumulative exposure to other stressors for fetuses, neonates, and mothers that is likely to have raised their allostatic load - the cumulative burden of chronic stress across physiological and psychological systems - in turn raising the likelihood of occult or frank disease, which may contribute to breastfeeding insufficiencies [172]. Modern changes contributing to such allostatic load increase include: environmental pollutant exposure [173], excessive hygiene that limits exposure to stimuli necessary for healthy immune development and function (the so-called hygiene hypothesis in allergy and immunology) [174], periodontal disease [175], suboptimal microbiome development and/or regulation [176], processed diet [177], physical inactivity [178], obesity [179], substance abuse [179], sleep impairments [179], social isolation [180], breakdown of social structures, increased reliance on different kinds of experts who made traditional support networks less relevant in combination with increased reliance on modern methods of measurement and other technologies that made habits such as eating and sleeping more quantified (as in the rise of "scientific motherhood" in the Progressive Era US [181], and continuing today with the evolution of the Internet of Things and the personal data revolution), and increased emphasis on the importance of individual choice in determining outcomes such as health outcomes, particularly when it comes to mothers and infants [182], amid unstable, atomistic, and structurally deteriorating conditions.

The modern Western condition links these possible allostatic load-increasing factors with the conditions that gave rise to the modern notion of "exclusive breastfeeding." New mothers supported by more experienced mothers with direct knowledge of safe breastfeeding, as in most traditional and non-Western societies, would not risk accidentally starving their newborns in the same way that new mothers without that human capital habitually do under the exclusive breastfeeding paradigm. Thus, intergenerational knowledge loss, the marginalization of laywomen as experts in childrearing and other forms of care work, and increasing social isolation all play a role in neonatal starvation from exclusive breastfeeding, as well as in other modern changes that may contribute to increased autism prevalence along other pathways.

Two additional possible contributing factors to rising autism prevalence do not quite so neatly fit this story, but also have to do with modernity in a broader sense. First, as Reser hypothesized, assortative mating may play a role in more severe autism, with solitary foraging conditions previously selecting for subclinical autistic traits and then modern systematizing thinkers (increasingly) selecting one another for mates [183]. Results from a Dutch school study by Roelfsema et al. are consistent with the assortative mating hypothesis, showing substantially higher autism incidence (229/10,000) in information technology hub Eindhoven as compared with other Dutch cities (Haarlem, 84/10,000; Utrecht, 57/10,000) [184]. Some interpret these data as evidence that modern mating may increase autism incidence. But these data could also be explained by the breastfeeding starvation hypothesis. Women in technological fields or their surrounding social settings could also (or instead) have more breastfeeding insufficiencies, e.g., due to metabolic problems from a sedentary lifestyle or environmental exposures; parents in these fields could have less of a tendency to have access to or to use informal knowledge and support networks for early infant care expertise, and/or more of a tendency to follow medical expert advice to exclusively breastfeed. So biological and behavioral predispositions alike could contribute to more exclusive breastfeeding-associated neonatal jaundice causing more autism in tech hubs.

Second, Rogers hypothesized that in the early 1990s in the US, increased folic acid intake among pregnant women that was intended to prevent neural tube defects resulted in an unintentional reduction of natural selection pressures against the polymorphic form of the 5-methylenetetrahydrofolate reductase (MTHFR) enzyme needed to activate folate for methylation in neurodevelopment, leading to an increase in *MTHFR* C677T polymorphic live births due to fewer miscarriages from hyperhomocysteinemia and associated thrombotic events, and thus an increased incidence of infants requiring additional and/or more bioavailable folate for normal methylation during the sensitive postnatal developmental period, resulting in increased autism incidence from subpar folate status [185]. If true, this folate activation selection hypothesis could interact with the breastfeeding starvation hypothesis, with compounding effects that might help explain some of the unexplained variations in clinical consequences of TSB measurements; greater folate need plus less overall nutrition could cause more harm in the affected subgroup.

Rogers' hypothesis, and its intersection with the breastfeeding starvation hypothesis, remains largely untested. A PubMed search for "*MTHFR* C677T TSB" returns no results (as of January 3, 2022); searching for *"MTHFR* C677T jaundice" revealed a case report and a sickle cell study; and searching for *"MTHFR* C677T neonatal screening meta-analysis" returns one meta-analysis by Wu et al., who found preterm birth and low birth weight associated with the polymorphism [186], and a spina bifida case-control study by de Franchis et al. that said the polymorphism is a risk factor [187]. In a study of over 7000 neonates from 16 worldwide areas, Wilcken et al. found marked geographical and ethnic variation of the polymorphism, with higher homozygous TT genotype frequency in particular in northern China, southern Italy, Mexico, and American Hispanic newborns, and low frequency among Africans [188]; this pattern does not match known geographic/racial jaundice-associated morbidity and mortality risks, which neither disproves nor supports the possibility that the mutation compounds risks from jaundice. Hence, modern supplementation may also play a role in increasing autism incidence, but the causal pathways through which that might be are numerous; more research is needed and available data do not match the interaction we might expect to see with breastfeeding insufficiencies if jaundice risks proxy for insufficient milk intake and increased folate need due to *MTHFR* C677T polymorphism compounds neurodevelopmental harm from starvation.

Elsabbagh et al. suggest that increased autism incidence is likely an artefact of social and administrative, rather than biological, changes [189]. This view is supported by Kujabi et al. and others. They hypothesize that changing diagnostic criteria, service and funding availability, and awareness cause increased autism prevalence. But the evidence is insufficient to disprove the possibility of a true increase, and the two possibilities are not mutually exclusive.

Nassar et al. suggested rising autism prevalence in Western Australia from 1983-1999 appears related to social and administrative changes but noted that a true increase cannot be ruled out [190]. In a systematic review and meta-analysis, Williams et al. estimated that differences in screening age, diagnostic criteria, and country account for most variation in autism prevalence study estimates [191]; but the authors do not appear to have considered that these variables could themselves reflect true incidence increase. For instance, the authors reported that "an increase of one year in the age of the children screened led to a significant reduction in prevalence estimates," a pattern consistent with possible increased autistic behaviors in younger children; this could reflect developmental lags relating to changes in early childcare practices (e.g., more screen time and less eye contact, touch, and holding) or early infant feeding practices (e.g., in the event of later sufficient nutrition, hydration, and time healing some reversible damage from neonatal deprivation). Similarly, experts could update diagnostic criteria to better reflect changing realities, and country-level variation could tend to covary with other factors possibly linked with a true increase, such as allostatic load, environmental pollutant exposure, or exclusive breastfeeding practices, in addition to potentially proxying for service availability or awareness.

Additional evidence for the social/administrative hypothesis of artefactual autism increase comes from two Scandinavian birth cohort studies, and this evidence is weak. First, in a Danish study of children born from 1980-1991, Hansen et al. found that diagnostic criteria changes and the inclusion of outpatient contacts (e.g., less severe cases) accounted for most of the increase [192]. But again, diagnostic criteria changes could reflect incidence changes; diagnostic criteria frequently change to better reflect evolving social and scientific knowledge. Additionally, some data suggest a decrease in more severe and an increase in less severe cases [190]; this is consistent with Hansen et al.’s inability to exclude moderate to mild case increase, which could reflect better prevention of progression and potential base rate increase. Second, in a population-based study of Swedish twins in the national patient register from 1993-2002, Lundström et al. observed that registered diagnoses increased while the autism symptom phenotype prevalence, as assessed by a validated parental phone interview, was stable [193]. While this finding is suggestive, the authors do not report the timing of the phone interviews or acknowledge likely relevant limitations. Retrospective self-reports are notoriously unreliable, and retrospective self-reports from 1993 in 2005 (for instance) may not be comparable to retrospective self-reports from 2002 in 2005.

Overall, the evidence suggesting no true rise in autism is weak; substantial autism prevalence increase cannot be dismissed as artefactual based on existing data. So how big is the increase?

**Figure 2:**
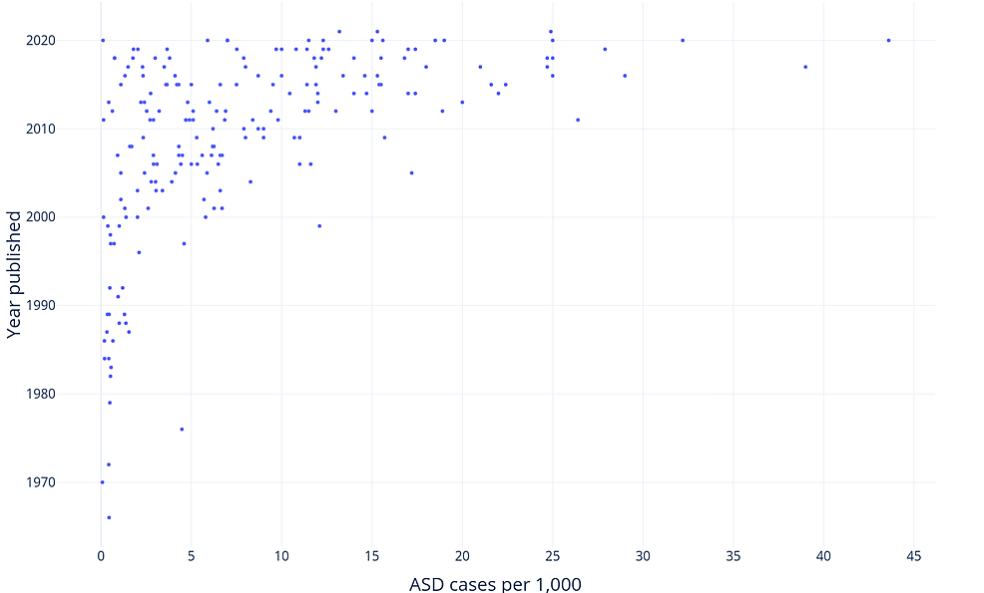
Estimated Autism Prevalence, 1970-2020 Data from the CDC autism prevalence study estimates collection [30]. ASD: autism spectrum disorder

As part of Public Health Surveillance in the National Center on Birth Defects and Developmental Disabilities, Child Development and Disability Branch, the US Centers for Disease Control and Prevention (CDC) maintains an autism prevalence study estimates collection. It tables information from English-language, peer-reviewed studies from PubMed searches for autism/autistic and prevalence (current through July 2021) that produce at least one autism prevalence estimate and are population-based within a defined geographic area. A scatterplot of that data (Figure 2) shows the generally accepted significantly increasing prevalence, with a more substantial increase since around 2000 and around 1-2% incidence.

This marked rise in autism [29,30] and other neurodevelopmental disorders [31] coincides with the introduction and rise of the exclusive breastfeeding paradigm [32-36]. There are many reasons to suspect the relationship is causal. Its modern Western creators imagined this new early infant feeding paradigm was natural. Ignorant of its historical novelty, they did not collect safety data on the intervention. Now, increasingly well-established possible mechanisms of harm include brain damage from hyperbilirubinemia caused or exacerbated by starvation/deprivation due to insufficient milk intake associated with exclusive breastfeeding.

The remainder of this section summarizes the state of the literature as it pertains to future research on two points. First, we need to hear more about the possibility that humans evolved not only as cooperative breeders [58,194], but also specifically as cooperative breastfeeders, and what that means for and about humanity biologically, psychosocially, and culturally. Second, we need to see better what the best available data can and cannot say about direct tests of the breastfeeding starvation hypothesis of increasing autism prevalence.

Historically, most prior societies practiced, and most non-Western societies continue to practice, widespread prelacteal feeding. In the vast majority of HRAF-documented foraging societies, this practice takes the form of shared nursing that prevents infants from going hungry in the days before new mothers' milk comes in. For instance, Hrdy notes that in both Efe and Aka tribes, another lactating woman from the tribe, or a wetnurse from a neighboring tribe, nurses the newborn during that period [58]. Also working from the HRAF, Post notes near-universal artificial feeding soon after birth [57]. Several scholars note widespread prohibition against infant consumption of colostrum (the thin first milk secreted before lactogenesis II/mature milk production) [37,195] - a taboo that, in Europe, persisted until the 1880s [196].

The sociality of early infant feeding in foraging and pre-modern societies is likely a survival response to evolutionary pressure from common breastfeeding insufficiencies that might otherwise substantially increase infant mortality and morbidity, particularly in the absence of modern medical care access. According to some estimates, first-time mothers are particularly prone to breastfeeding insufficiencies, with 33-44% perceiving milk coming in (lactogenesis II) beyond 72 hours postpartum [9,10] and 15% still producing insufficient breastmilk after three weeks despite intensive professional lactation support [6]. It would not have made sense for our ancestors to let one in three firstborns risk death or permanent disability from several days of avoidable starvation - nor for them to conceptualize early infant feeding as uniquely the responsibility of the mother when other aspects of childcare, including provisioning, probably tended to be shared among multiple caregivers.

Pre-modern organized societies that left sufficient historical records for this type of investigation appear to have similarly maintained safety infrastructure to protect newborns from common breastfeeding insufficiencies. Wetnursing [37-41], co-nursing [42], and prelacteal feeding [43-56] were widespread and well-ordered early infant feeding practices. Today, this safety infrastructure persists in most of the non-Western world, including among the vast majority of foraging societies documented in the HRAF [58]. In most societies in the HRAF and Standard Cross-Cultural Sample, mothers initiate breastfeeding at least a day and up to a week postpartum, in contrast with the modern, Western norm of immediate postpartum breastfeeding initiation. Where such prelacteal feeding and shared nursing norms had been largely replaced by modern formula-feeding, the subsequent introduction of "exclusive breastfeeding" without an attendant safety infrastructure addressing common insufficiencies led to an epidemic of common and preventable harm to neonates from insufficient nutrition/hydration [26,27,59-67]. Modern disconnection from previous care work knowledge enabled this tragic accident. Before its relatively recent resurgence, the pendulum had swung away from breastfeeding in many modern, Western societies for generations. This created intergenerational knowledge gaps about safe breastfeeding. Well-meaning reformers then brought breastfeeding back with a new, historically anomalous emphasis on exclusivity, and in the absence of the pre-existing safety infrastructure. So unintentional, epidemic harm from intentional neonatal starvation is a uniquely modern, Western problem which has been assiduously exported worldwide by well-meaning reformers.

Why are breastfeeding insufficiencies so common if they are an antecedent condition of so much neonatal harm? The fact that homo sapiens tend to be a slow-maturing species offers one plausible sort of explanation. The commonality of breastfeeding insufficiencies, particularly among first-time mothers, is likely due physiologically to lack of full breast tissue differentiation during adolescence and first pregnancy; human breast tissue continues differentiating as a woman continues maturing in her reproductive life through childbearing, with mammary gland differentiation typical of pregnancy (causing the usual associated breast changes, which are predictive of functional lactation [6]). Slow maturation is one facet of our costly reproduction (and vice-versa). Another is that gestation and birth use finite maternal bodily resources that other mammals with less expensive gestation and birth may retain for faster, less failure-prone lactation. But while these explanations may account for greater primapara incidence of breastfeeding insufficiencies, it is unclear that they fully explain the provenance or function of a days-long period before milk comes in for most human mothers. Is this typical of other mammals, particularly primates?

Hinde and Milligan observed that little is known about changes in primate milk synthesis across lactation, information that seems relevant to answering this question [197]. Urashima et al. found differences in the colostrum of humans, primates, and other mammals with human milk containing mostly type I over type II oligosaccharides; type II predominating in chimpanzee, bonobo, and orangutan, and exclusively in gorilla and siamang milk as well as almost always in nonprimate milk [198]. So it appears that human colostrum is situated at one pole of a continuum of different milks in terms of this compositional aspect. Urishima et al. note that type I human milk oligosaccharides (HMO) likely support infant gut colonization by beneficial bifidobacteria, concluding "The biological importance of type I HMO predominance warrants further study, both in relation to human health and to human evolution" [199]. A wide array of evidence suggests HMO contributes to optimal microbiome development along multiple pathways, exerting antimicrobial, prebiotic, mucosal barrier maturation, and immune system developing effects [200]. So might type I HMO be more costly for lactating mothers to produce, but offer their neonates compensatory benefits, partly explaining possible differences in early milk production between humans and other mammals that necessitated or reinforced humans' evolution as cooperative breeders in general and cooperative breastfeeders in particular? Or is something else going on that makes modern human lactation particularly failure-prone?

One alternate, though not mutually exclusive, explanation is that lessened selection pressures on functioning lactation for infant survival in herding societies may have caused women in those societies, over generations, to disproportionately lose breastfeeding as a reliable function. Levin posits and Post builds on this hypothesis [196,57]. Both assume that feeding infants other milks is almost unique to Caucasians and their ancestors. But the literature subsequently widely notes that lactose tolerance (also known as lactase persistence or lactose absorption versus malabsorption) persists into adulthood in some African and Asian populations, too [201-3]. In an analysis of all published relevant data including data on adult lactose tolerance, Sherman and Bloom find that in 270 populations, it is associated with favorable historical conditions for dairy herding, namely, less extreme climates (moderate latitudes), and absence of historical (pre-1900) cattle diseases [201]. So North African [202] and East African [203] but not West African populations frequently exhibit lactose tolerance that seems to have evolved due to ancestral dairy consumption patterns, as do their Scandinavian and other northern European, historically dairy-farming counterparts. Nomads who could migrate with their herds to avoid weather extremes also frequently have it, while diasporic populations without a history of ancestral dairy herding practices (e.g., Jews) lack it. This diversity may make it easier for future research to test the decreasing selection pressure hypothesis of increasing breastfeeding insufficiencies. On one hand, data are unavailable on breastfeeding insufficiencies across racial subgroups over time, the ideal data for such tests. On the other hand, correlation between lactose tolerance and higher base rates of breastfeeding insufficiencies across diverse groups would be suggestive of support for this hypothesis, and the latter data could be collected using maternal self-reports.

Less selection pressure might help explain more lactation problems in evolutionary time, but it is also possible that novel modern conditions cause marked increases in the frequency and severity of breastfeeding insufficiencies. Increasing exposure to environmental pollutants is linked with the increasing incidence of numerous fertility and other health problems in animals including humans - including ones associated with breastfeeding problems, such as PCOS, overweight/obesity, and diabetes. Such exposures might drive a rising incidence of breastfeeding insufficiencies, though again, data do not exist to establish base rates of such problems over time to measure changes in them.

For instance, dioxin exposure during pregnancy impairs murine mammary gland differentiation, blocking lactation [204] by activating the aryl hydrocarbon receptor (AHR) [205] (and thus starving the pups to death within a day). This pollution is prevalent, affecting women worldwide. Timmermann et al. established the generalizability of such pollution-undermined lactation to humans through their studies involving two Faroese birth cohorts in which maternal serum persistent perfluoroalkyl substances (PFASs) reduced breastfeeding exclusivity and duration [206] (Faroe Islanders have long been the subject of special attention in environmental pollution exposure research due to their consumption of pilot whale containing marine toxicants). Different country-level environmental regulation regimes and population-level exposures may help explain different base rates of neonatal jaundice, although again, data on the breastfeeding insufficiencies that would causally link the two do not exist; future research should assess the relationship between dioxin pollution on one hand, and neonatal jaundice and other potential complications of breastfeeding insufficiencies on the other. Environmental pollution may increase not only breastfeeding insufficiencies but also second-hand exposure to toxins stored in fatty breast tissue and passed on through breastmilk to infants during the sensitive developmental period after birth.

A growing body of evidence shows that infants do ingest pollutants through breastmilk, and these exposures appear to adversely affect development; so it could be a protective mechanism that some toxins short-circuit lactation, as long as offspring have an alternate source of sustenance. Analyzing Faroese birth cohort serum evidence, Mogensen et al. showed breastfeeding duration and exclusivity are associated with concentration increases of most PFASs in children [207]. Grandjean et al. calculated that transfer of contaminants from human milk fully explained the slowed postnatal growth of breastfed infants (a typical pattern compared to formula-fed infants) in a term Faroese birth cohort from 1994-1995 [208]. In later research, Grandjean et al. showed PFAS exposures in infancy are associated with attenuated tetanus and diphtheria vaccine antibody responses at age five, with these persistent immunotoxic effects most affected by exposures during the first six months postpartum, when breastfeeding drives increased PFAS exposures [209].

If the transfer of environmental pollutants to infants through breastmilk may cause pediatric harm, then it should also benefit mothers by offloading those otherwise persistent toxins; and there is evidence that it may. Examining PFAS exposures and type 2 diabetes incidence in a case-control study of women from the US Nurses' Health Study II, Sun et al. found PFAS exposures in the late 1990s are associated with increased later diabetes risk, consistent with a potential diabetogenic effect of PFAS exposures [210]. Analyzing the same data with an analogous hypothesis, Zong et al. found exposure to persistent organic pollutants (POPs) more broadly may also be diabetogenic [211]. These links are consistent with the observation by Nommsen-Rivers et al. that disturbances in maternal glucose metabolism may link several risk factors for breastfeeding insufficiencies: pollutants may impair lactation through pathways that also have negative metabolic consequences for women later, with weight gain increasing both pollutant load and metabolic problems in a vicious cycle, as more fat stores more fat-soluble toxins and both harm glucose metabolism [10]. Similarly, Brody and Rudel suggest that maternal elimination of carcinogenic substances through breastfeeding could contribute to an apparent inverse association between lactation and breast cancer risk [212]; this would mean that women lower their breast cancer risk by transferring carcinogenic substances to their infants through breastmilk.

Swan and Colino offer a framework to situate such concerns in the broader context of growing evidence that male and female reproductive development, behavior, and function are increasingly and substantially impaired by environmental pollution [213]. So modern breastfeeding insufficiencies, and the potential risks of environmental pollutant transfer that they implicate, may to some extent be as historically anomalous as the modern emphasis on breastfeeding exclusivity, which endangers infants. But for all our modern drive to data collection, the absence of systematic evidence on lactation problems limits relevant hypothesis testing. This absence of evidence is a legacy of modern Western doctors and researchers not believing the substantial minority of women who have long reported breastfeeding problems including insufficient milk. We do know, however, that most mothers' mature milk has always taken days to come in, and that only the current early infant feeding paradigm advises "exclusive breastfeeding" in those days.

What does all this mean in terms of the evolutionary puzzle that common breastfeeding insufficiencies pose? When, why, and how did humans evolve as not only cooperative breeders, but also specifically as cooperative breastfeeders? Were breastfeeding insufficiencies primarily an accident of biology, e.g., of the costliness of reproduction in our slow-maturing species? Or were common breastfeeding insufficiencies adaptive in evolving humans as cooperative breeders? Pre-human and early human mothers, especially first-time mothers, likely to physically require the assistance of more experienced mothers in order to feed their babies initially may well have benefited in other ways from proximity to such expert help. So, too, might their offspring and alloparental support networks. Conversely, such advantages would imply possible disadvantages from current, relatively atomistic, modern Western early infant feeding and other care norms - norms that many criticize as contributing to postpartum mental health problems. Future research should review evidence relevant to this puzzle.

Historically and culturally, the real puzzle is not why humanity before modern Western society generally shared certain infant feeding norms, but rather why modern Western reformers changed those norms radically and without evidence establishing their safety, running a global natural experiment in the absence of informed consent or data collection about its possible risks. Aware that they were missing generations of knowledge about early infant feeding, reformers constructed a new, historically anomalous set of breastfeeding practices that introduced a single point of failure (one mother's lactation) into what had previously been a cooperative enterprise. Future research should examine the question of why modernity atomizes in this context, assessing to what extent this story is sui generis. Where else have modern societies marginalized, devalued, and lost traditional care work knowledge, and with what consequences for health? The rapid global loss of linguistic diversity, traditional diets, and ecosystems comes to mind.

Moving back down again from the evolutionary to the mechanistic level, it would be ideal to directly test the breastfeeding starvation hypothesis of increasing autism prevalence. But, because of a lack of systematic data collection on relevant variables such as neonatal starvation periods, breastfeeding problems including maternal perceptions of delayed and insufficient milk, and heterogeneity in implementation of exclusive breastfeeding policies, the data for these tests do not appear to exist. This heterogeneity is notable: Hofvander reports that Swedish Baby-Friendly hospitals gave healthy neonates prelacteal feedings (i.e., formula before their mother's milk came in) anywhere from under 10% to 90% of the time [214]; implementation of the exclusive breastfeeding paradigm seems to have been highly heterogeneous and rarely documented in relevant detail. Lipsky notes public service professionals interacting directly with citizens in the course of their jobs, like teachers and police officers, often have substantial discretion in executing their work, terming their apparatus street-level bureaucracy; it is this bureaucracy that determines what policies really mean in practice [215]. The tremendous variation in what exclusive breastfeeding means for babies in terms of starvation/deprivation exposure could similarly be thought of as an example of street-level health policy, a type of phenomenon often not measured by quantitative datasets. Thus, existing evidence does not appear to permit direct tests of the hypothesis that exclusive breastfeeding caused increased starvation that increased hyperbilirubinemia incidence and thus autism rates through associated permanent brain damage. But future research should investigate whether such tests are indeed possible, for instance using a more granular form of the sort of Swedish hospital-level data that Hofvander cites. It may also be worthwhile to compile published prevalence data on breastfeeding, exclusive breastfeeding, and the exclusive breastfeeding paradigm, in spite of the apparently substantial but largely invisible heterogeneity in what is being measured vis-a-vis neonatal starvation. The ideal dataset would collect data on global maternal perceptions of breastfeeding insufficiencies, early infant feeding behaviors, and subsequent neonatal complications and pediatric outcomes in order to estimate effects of neonatal starvation; but this dataset does not exist and would not be ethical to create, because neonatal starvation is trivially treatable and already known to pose substantial possible risks.

Neonatal jaundice incidence presents another set of measurement difficulties in relation to direct tests of the breastfeeding starvation hypothesis of increasing autism prevalence. As previously noted, under-diagnosis despite heightened associated risks in darker-skinned infants may persist for several reasons; related error rates are likely to have changed over time as this issue has garnered greater attention and understanding. Diagnostic criteria changes present another challenge, as do differences in measurement, severity, duration, standard use of TSB instead of unconjugated bilirubin (discussed in greater detail later), and growing normalization of related problems as the exclusive breastfeeding paradigm gained dominance. Future research should note these sorts of limitations and synthesize published neonatal jaundice incidence data with the aim of seeing if the base rate appears to have increased.

It is past time to put the burden of proof where it belongs: Existing evidence is insufficient to establish the safety of days-long neonatal deprivation/starvation with respect to children's neurodevelopmental outcomes. The burden of proof should be on the side of establishing the novel modern medical intervention, exclusive breastfeeding, as safe. Research and practice must honor the Hippocratic Oath by focusing on first preventing harm when this intervention results in lengthy periods of insufficient milk intake for neonates, which carry serious possible risks.

If modern exclusive breastfeeding at least partly caused increased autism prevalence via neonatal neurological damage from deprivation, then we might think of the shift in early infant feeding norms that the new construction of breastfeeding introduced as a natural experiment. We would expect to see worse outcomes in the treatment than in the control group. The evidence fits: the apparent paradox of weakened preterm jaundice-autism (and related) associations makes sense in the context of different subgroup feeding norms among other factors. Preterms may be the control group in this natural experiment.

The preterm paradox: a natural experiment in preventive nutrition?

Preterm subgroup results from the jaundice-autism literature present an apparent paradox. While substantially more vulnerable to morbidity and mortality, including jaundice [142] and autism [216], preterms appear unexpectedly less vulnerable to the considerable possible jaundice-autism effect. All three meta-analyses showed weaker associations in this subgroup, with confidence intervals estimating possible risk increases up to 2%, 13%, and 12%, respectively, and the two that reported statistical significance in the aggregate (Amin et al. and Jenabi et al.) lost it in this subgroup analysis.

Amin et al. estimated a subgroup OR of .7 (95%CI: 0.38-1.02) and suggested it still "deserves further investigation" because it was "contrary to our hypothesis and biological plausibility." They hypothesized it might be due to lower protein concentration and altered binding affinity due to competitors with bilirubin for albumin binding causing high (neurotoxic) free unconjugated bilirubin concentration despite low TSB concentration in preterms. In a later review of the evidence on unconjugated hyperbilirubinemia's developmental influence on neurobehavioral disorders, Amin noted, "Limited data is available on the ontogeny of (bilirubin binding capacity) and affinity in premature infants" [217]. Similarly, Jenabi et al. estimated an OR of .89 (95%CI: 0.65-1.13), echoing Amin et al.’s observation that this puzzlingly weakened preterm subgroup finding might be explained by a high concentration of (neurotoxic) free unconjugated bilirubin despite low TSB due to physiological differences in this subgroup. Kujabi et al. found a preterm OR of .93 (95%CI: 0.77-1.12) and noted that preterms were more often subject to hospitalization. This makes them more likely to be diagnosed with jaundice, which the authors observed could drive a spurious correlation; but this conflicts with the idea that damage may be done by the time of jaundice diagnosis.

Three factors may help explain this apparent paradox. First, preterm subgroup estimates are particularly vulnerable to downward bias. Underpowering caused by small sample size, narrow operationalization of the outcome of interest (focusing on autism to the exclusion of other risks), and survivorship bias are particularly likely to contribute to Type-II errors (finding no significant effect when there is one) in preterms, a minority of births with considerably higher morbidity and mortality risks. Walani notes preterm birth is the leading cause of death among children, with substantial variations in preterm birth rates and associated mortality between and within countries [218]. Thus, heightened mortality and relatively severe morbidity among jaundiced preterms may contribute to the appearance of a weakened jaundice-autism effect in this subgroup, while truly reflecting an even stronger relationship between neonatal jaundice and practically significant, preventable harm. Because preterm neonates are more biologically vulnerable in many contexts, they may have higher rates of more severe jaundice-associated disability (e.g., kernicterus) and death (e.g., from acute bilirubin encephalopathy or ABE) than their near- and full-term counterparts, particularly within the most vulnerable subgroups - that is, among very low birth weight infants, those with co-morbidities such as birth trauma or infection, and in lower-resourced settings.

Second, preterms have heightened exposure to cumulative perinatal risk factors for autism. This heightened exposure to other risk factors may make jaundice appear less impactful in this subgroup, because susceptible children's genes or other predisposing factors in prodromal syndromes could have already been activated in relevant ways. In a review of evidence on environmental influences on autism development, Mandy and Lai suggest gene-environment interplay influences the development of autism spectrum condition, with postnatal social factors influencing the likelihood of prodromal autism progressing [219]. This logic extends the biological and meta-analytical findings others report, that cumulative prenatal and perinatal adverse event exposure may increase autism risk.

For instance, Chien et al. reported that autistic children had higher numbers of prenatal/perinatal factors that may increase the risk of ASD as compared to both their unaffected siblings and typically developing controls; particularly severe autism symptoms were associated with preeclampsia, polyhydramnios, oligoamnios, placenta previa, umbilical cord knot, and gestational diabetes [220]. In a twin study, Froelich-Santino et al. reported that factors associated with respiratory distress and hypoxia increased autism risk in males, while jaundice increased it in females [106]. This was a counter-intuitive finding, because a wide array of evidence suggests male gender is a risk factor for greater susceptibility to harm relating to nutritional scarcity [221]. This finding is consistent, however, with the hypothesis that innate susceptibility combines with the accumulation of adverse event exposures during sensitive developmental periods, causing jaundice to increase autism risk more in these twin females due to their lower vulnerability to problems associated with early nutritional stress in general and lower susceptibility to autism in particular; maybe the predisposed males' autism was already activated. In a review of the literature on pre-, peri- and neonatal risk factors for autism, Guinchat et al. posited that such factors could be "causal or play a secondary role in shaping clinical expression in individuals with genetic vulnerability," especially considering how "improvements in obstetric and neonatal management have led to an increased rate of survivors with pre-existing brain damage," emphasizing the importance of investigating multifactorial stories [222].

Together, findings like these provide some preliminary support for the hypothesis of a threshold of cumulative adverse event exposure, perhaps acting on more susceptible children to increase autism risk such that not all exposures affect all children equally. This hypothesis warrants further investigation and may help explain the weakened jaundice-autism preterm effect. With power limitations, it may be testable with meta-analyses exploring subgroup effects within the preterm subgroup. For instance, we might expect a notably weakening male subgroup effect for progressively later- rather than earlier-occurring groupings of cumulative adverse event independent variables, because males tend to have a greater susceptibility to most relevant variables and so we might expect the effect of cumulative adverse event exposure to be greater on them, sooner.

Third, the preterm paradox may also (simultaneously) reflect a true difference in jaundice-autism risk due to more intensive early intervention that improves nutrition, limiting jaundice progression and duration. Heightened monitoring likely results in faster diagnosis and treatment for preterms who develop jaundice, hypoglycemia, and/or hypernatremic dehydration - frequent complications of insufficient milk intake associated with breastfeeding that cause most of the approximately 80,000 annual US neonatal hospital readmissions [4,5]. Different norms surrounding supplementation in preterms also improve nutrition: preterms enjoy preferential access to limited, banked donor breastmilk, are typically fed with fortifiers regardless of milk type (increasing milk's nutritional value in terms of calories and vitamins/minerals), and are more often fed formula due to broad acceptance of medical necessity, with insufficient breastmilk widely acknowledged as common among mothers of preterms (but not among mothers of near- or full-terms, despite its commonality across groups). So weaker preterm jaundice-autism associations are consistent with the link between insufficient milk intake associated with exclusive breastfeeding, and preventable harm. This explanation is furthermore consistent with what we know about jaundice and feeding mode: Jaundice occurs rarely in formula-fed infants and frequently in breastfed ones due to insufficient milk intake [223]. Preterms screened earlier for jaundice and fed formula more frequently will tend to resolve the problem faster, and prevent its progression more aggressively, than their later-born counterparts.

In this way, the preterm paradox may reflect results of a natural experiment in preventive nutrition. While healthy near- and full-term infants act as the experimental group in the modern "exclusive breastfeeding" experiment, preterms are the control group. The control group fares considerably better when it comes to increased autism risk associated with jaundice. This is consistent with analogous findings that neonatal hypoglycemia increases term infants' autism risk threefold but does not increase the risk in preterms [224] - although such findings are also consistent with the other possible factors explored above including underpowering, survivorship bias, and an already activating threshold of cumulative adverse event exposure for predisposed people. The natural experiment story is also consistent with the idea that time matters and associated harm is preventable. The mechanisms of harm from insufficient nutrition and hydration progress over time, with jaundice worsening as bilirubin builds up with significantly decreased voiding in breast- versus formula-fed infants [225].

So the preterm puzzle fits the breastfeeding insufficiencies story, but what else do we know about how medical management influences jaundice outcomes with respect to autism risk? Does phototherapy, the current standard treatment for neonatal jaundice that is determined severe enough to require medical intervention, mitigate the risk? The body of evidence on this question presents an under-recognized puzzle, and a parable in the wisdom of scientific modesty.

Phototherapy and autism: we don't know what we think we know

A few generations ago, the standard medical treatment for jaundice in breastfed neonates was switching them to formula. Then modern reformers brought back breastfeeding with a historically anomalous emphasis on exclusivity. Around that time, phototherapy replaced formula-feeding as the standard treatment for neonatal jaundice severe enough to require medical intervention but not severe enough to require the more invasive and riskier hospital treatment of exchange transfusion. But the evidence for this shift was limited. Safety concerns plague current practice. This matters because it underscores the wisdom of applying the precautionary principle by treating the root cause of modal neonatal jaundice, insufficient milk intake associated with breastfeeding, by returning to previously widespread early infant feeding norms centered on ensuring that newborns drink enough milk.

Many researchers caution that phototherapy may cause harm [226] including activating pro-inflammatory gene expression [227] with neuroinflammatory effects [228-230], increased epilepsy risk [231], immune modulation and associated increased allergic disease [232-234], DNA damage [235] and associated cancer [236,237], and increased extremely low birth weight neonate (ELBWN) mortality [238]. Limited research addresses the question of phototherapy duration and potential iatrogenesis. Ahmed et al. [109] reported that mean days of phototherapy predicts autism, and the difference is large in mean days (17.68 ± 5.946 for the autism group versus 1.43 ± 3.163 for the control group) and highly statistically significant (p = .0002). But jaundice duration or persistent insufficient milk intake could also explain the effect, and both remain understudied. The evidence is inconclusive, but phototherapy is a potential confounding variable for neurodevelopmental and other associated harm, including autism, in jaundiced neonates.

We might see correlations between phototherapy and worse outcomes for four reasons, and they are not mutually exclusive. First, jaundice severity/duration could predict phototherapy treatment, with one or both of those facets of jaundice (in combination with other risk factors) doing the harm. More severely jaundiced neonates are probably more likely to both receive phototherapy treatment for hyperbilirubinemia and to develop later autism as a result of bilirubin-induced brain injury. Duration, an under-studied factor, offers a particularly promising explanation for why even studies that account for jaundice severity may show increased autism risk associated with phototherapy; bilirubin-induced brain injury, with time as a critical factor, could explain irreversible neurological harm. Second, other factors including prematurity and infection could drive jaundice severity/duration, phototherapy exposure, and associated risk. But while jaundice duration is not usually reported and so not included in relevant analyses, preterms are usually identified and analyzed separately due to widespread recognition of increased vulnerability, making this type of explanation less likely. Third, phototherapy could cause iatrogenic harm through posited mechanisms including pro-inflammatory gene expression, incurring additional risks on top of damage jaundice does by the time of diagnosis. This could be largely masked by jaundice and other comorbidities; if phototherapy heightens a spectrum of pro-inflammatory risks, but children already more vulnerable to such risks are disproportionately treated with phototherapy in the first place, then evidence of such harm could be wrongly dismissed on the basis of analyses that appear to correct for confounding variables. Fourth, phototherapy treats jaundice but not other complications of insufficient milk intake including hypoglycemia and hypernatremia; yet it replaced the prior standard treatment of switching jaundiced, breastfed neonates to formula. Thus phototherapy may proxy for untreated insufficient milk intake, other complications of which also risk possible permanent neurological damage and could also explain an apparent phototherapy-autism effect.

There is some evidence that phototherapy depends on sufficient milk intake for its efficacy and possibly safety. McDonagh notes that the effect of phototherapy hinges on excretion: "The principal effect of the treatment is not photodegradation of bilirubin, but the conversion of the pigment to structural isomers that are more polar and more readily excreted than the normal, more toxic 'dark' form of the pigment. This, coupled with some photooxidation of bilirubin, diminishes the overall pool of bilirubin in the body and lowers plasma levels" [239]. Thus, phototherapy might be more effective and/or safer when combined with formula supplementation, which speeds excretion. In line with this possibility, De Carvalho et al. showed that formula-fed infants excrete significantly more bilirubin through voiding (and thus overall) compared with breastfed infants [225]. Ma and Fan showed that low-birthweight preterms who received earlier nutrient supplementation exhibited improved jaundice outcomes, probably due to enhanced bilirubin excretion/elimination [240]. So it appears that insufficient milk intake first contributes to jaundice and its progression, and then compromises phototherapy efficacy; despite this, the effects of formula supplementation on phototherapy safety do not appear to have been studied.

There appear to be no published meta-analyses on phototherapy and autism or other neurodevelopmental harm along the potentially related continuum of the BIND spectrum that Amin et al.'s review detailed [28]. PubMed returns no results for the combined search terms "meta-analysis phototherapy autism" (as of February 4, 2022). Results for "meta-analysis phototherapy neonatal jaundice" similarly indicate no systematic study of the literature on the BIND spectrum in this context.

There are two clues in the jaundice-autism meta-analytical literature that phototherapy treatment for neonatal jaundice may risk increased autism. First, Amin et al. noted that, according to Sugie et al. [96], jaundice defined as need of phototherapy risks autism more than jaundice defined as degree of hyperbilirubinemia. This is consistent with the possibility that phototherapy independently contributes to increased autism risk, although the contribution could be correlational (as in the case of persistent insufficient milk intake) as opposed to causal (as in the case of pro-inflammatory gene expression). Second, Kujabi et al. observed that autism diagnosis has increased substantially in the same period that updated hyperbilirubinemia diagnostic guidelines increased neonatal jaundice admissions; this might suggest that the current treatment does more harm than good. So what does the underlying literature show about the increased risk of autism associated with phototherapy as compared to the increased autism risk associated with jaundice?

Results from the six studies included in the most recent jaundice-autism meta-analysis, Kujabi et al's [3], that also include phototherapy results are consistent with the possibility of a phototherapy-autism link. Chen et al. reported a considerably higher possible risk increase associated with phototherapy (HR 1.82, 95%CI: 0.79-4.20) than with jaundice (HR 1.75, 95%CI: 1.05-2.90) [101]. Chien et al. used phototherapy as their jaundice definition and reported an OR of 1.42 with 95%CI 0.79-2.56 in a sibling-control study - suggesting a substantial possible risk increase of up to 156% [220]. Lozada et al. reported jaundice OR of 1.18 (95%CI: 1.06-1.31) versus phototherapy OR of 1.33 (95%CI: 1.04-1.69) [104]. Sugie et al. did not report confidence intervals, but their statistical significance test results indicate that the estimated intervals do not overlap zero for the proportion of the autism group with hyperbilirubinemia (p < 0.05) or phototherapy (p < 0.0005) [96]. Both effects are statistically significant, and the effect of phototherapy is two orders of magnitude more highly significant; however, relative effect magnitude is what we care about here, the authors did not report it, and attempts to reach them for clarification were unsuccessful. Wu et al. also estimated substantial possible associations between jaundice and autism (RR 1.4, 95%CI: 1.1-1.6) and phototherapy and autism (RR 1.7, 95%CI: 1.5-1.8); both lose statistical significance (dropping to HR .89-1.35 and .98-1.24, respectively) after adjustment for confounds, but sizeable possible effects remain and cannot be dismissed [241]. The phototherapy effect here loses "significance" on the basis of a few hundredths of a point in the estimate. Of this subgroup of included studies that report phototherapy effect estimates, only Maimburg et al. reported lower autism effect estimates for phototherapy (OR 3.3, 95%CI: 1.0-10.1) than for jaundice (OR 3.7, 95%CI: 1.3-10.5) [97]; the difference is negligible and the possible effects substantial.

Overall, these sets of estimates substantially overlap, reflecting possibly crucial effects that warrant careful consideration and further research. This evidence troubles the notion that phototherapy protects infants from harm. Rather, it suggests that phototherapy for jaundice may be associated with even more preventable harm, in terms of later autism risk, than jaundice itself. This is a major unreported finding of reported data. Why might it have been missed?

A benign possible explanation centers on the common misuse of statistical significance tests as tests of practical significance. A less benign one centers on hospital systems making more money treating neonatal jaundice with phototherapy than they would preventing and treating insufficient milk intake with formula supplementation - and many also standing to lose additional funding for promoting the exclusive breastfeeding paradigm, should they depart from it. These explanations are not mutually exclusive. This review focuses on statistics. Future research should investigate the financial stakes, and how they relate to other sources of possible bias including not only perverse incentives in the classical understanding, but also cognitive and emotional, social and political, and normative and institutional factors and forces.

So how often do researchers in this field confuse statistical for practical significance, and what are the implications of this confusion? In these six phototherapy-autism studies, half (three) have confidence intervals overlapping zero, so making this mistake is possible; of those, most (two) make it. Wu et al. concluded "After adjustment for the effects of sociodemographic factors and birth weight, neither hyperbilirubinemia nor phototherapy was an independent risk factor for ASD," even though their confidence intervals estimated substantial possible risk increases (estimating upper bounds on hyperbilirubinemia and phototherapy multivariate HRs 1.35 and 1.24, respectively [241]). Similarly, Chen et al. [101] wrongly dismissed the phototherapy effect that their analysis estimated, which is of a larger magnitude than the jaundice effect they estimated, because the former's confidence intervals overlap zero while the latter's do not. But they reported results of multivariate Cox regression models showing substantial possible neurodevelopmental risks associated with phototherapy after correction for gender, urbanization level, and comorbidities: effect estimates for autism (HR 1.82, 95%CI: 0.79-4.20), ADHD (HR 0.97, 95%CI: 0.67-1.39), any developmental delay (HR 1.15, 95%CI: 0.84-1.57), developmental speech or language disorder (HR 1.41, 95%CI: 0.95-2.09), developmental coordination disorder (HR 1.63, 95%CI: 0.69-3.85), and intellectual disability (HR 1.10, 95%CI: 0.60-2.01) all include practically significant possible risks. So while they correctly concluded based on their analysis that "Newborn exposure to hyperbilirubinemia was related to the increased risk of developing ASD, any developmental delay, and developmental speech or language disorder in later life," they might have extended this conclusion to possible larger increased risks associated with phototherapy. So the mistake is common in the literature, and its implications for protecting (or failing to protect) newborns may be quite grave. Is this unusual?

Recently, the commonality and gravity of such misinterpretation of statistical tests [242-245] motivated leading statisticians to call en masse [246,247] for the dichotomizing concept - statistically significant or not according to a threshold, usually p ≤ 0.05 - to be abandoned, because it leads to hyping some claims while dismissing other, possibly crucial effects, on the basis of misinterpreted evidence. These criticisms are longstanding and generally accepted by leading statisticians and other experts across the scientific community.

But while banning p-value thresholding offers one simple solution, statistical significance testing misuse is not merely a technical or linguistic problem; it also has cognitive, social, and epistemological roots and implications. Greenland suggests that a cognitive bias of "dichotomania" drives misinterpretation of uncertainty as certainty in dismissing potentially practically significant but statistically non-significant results; statistics education, he argues, should address such biases [248]. Ziliak and McCloskey reviewed a number of examples before noting "The history of this persistent but mistaken practice (of relying on statistical significance test results as a heuristic for practical significance) is a social study of science" that might be understood variously as "'trained incapacity" (Thorstein Veblen), " 'the bureaucratization of knowledge'" (Robert Merton), and "the 'scientific prejudice'" (Friedrich Hayek) [249]. Going forward, Gelman proposes moving "toward a greater acceptance of uncertainty and embracing of variation," as opposed to merely reforming misuse of p-value thresholds [250].

Such calls to epistemic reorientation have considerable social and political significance. They resonate with Oreskes and Conway's analysis of how wealthy, powerful industries from tobacco to oil have misused uncertainty to misrepresent evidence on serious health risks that threatened their financial interests [251]. Scientists need to communicate better about uncertainty and what it means in terms of harm prevention. This challenge is heightened by common cognitive biases, bad professional norms, and organized opposition from powerful interests. Failing to surmount it has already cost innumerable lives. Greater emphasis on possible effects and their practical meaning, beyond significance test results, is widely viewed as a new and improved standard among methodologists. But this recommended shift has yet to percolate into common practice in the medical or scientific literature. So why do most journals, reviewers, and scholars ignore the change?

The simplicity of the technical change belies its implementation’s cognitive and emotional complexity, as it entails adopting a different approach to uncertainty. In publications, it requires shifting substantive results reporting focus from binary statistical significance test results to the practical significance of findings, incorporating uncertainty as part of the actionable scientific picture. This may involve moving from narrow "normal science" puzzle-solving in the Kuhnian sense, to bigger-picture questions about what would make the world better. This work is worth doing, but it takes a lot of (unpaid) work; uncertainty can and should be used to invoke the precautionary principle in the public interest, not only to discredit scientific evidence that threatens the financial interests of the powerful. "Publish or perish" remains the most oft-cited reason why this work remains largely undone, but only naming the problem risks feeding a vicious cycle of alienation and bad science. The political implications of changing this system have yet to be fully articulated or harnessed for change. We should talk more about why critically reviewing statistical methodology is political, and embracing uncertainty is a way of resisting illegitimate authority. Could more institutional support for partnerships between methodology, subject-area, and strategic litigation experts help advance this shift? How might methods training and open-access publishing help experts who are outsiders, like grassroots activists and traditionally disadvantaged stakeholders, advance their causes? How do courts, legislatures, and the public react when "the little guy" uses (among the more reputable) industry tactics and empiricist tools to serve under-served communities? The social and political impact of the recommended shift from statistical to practical significance remains to be shaped by the scholars and institutions struggling to address the crisis in science, and more awareness in shaping it could generate more momentum to help address it. 

One thing is clear: implementing the indicated epistemic shift toward embracing uncertainty suggests a need for better safety data on phototherapy treatment for neonatal jaundice. Future research should better assess whether and how phototherapy might contribute to neurodevelopmental and other risks in formerly jaundiced neonates. Methodological considerations relevant to the implicit question of interaction suggest this research should be experimental. Greenland cautions that "even with huge effects and perfect data... identification of specific mechanisms of interaction will depend on biologic assumptions that are untestable (nonidentifiable) with epidemiologic observations alone" [252]. We need to know how supplementation and phototherapy interact. We also need to know how hospital systems respond to financial incentives to treat neonatal jaundice with in-patient phototherapy rather than preventing it with formula supplementation, another question arguably best addressed through experiments; health insurance companies can measure the effects of experimental reimbursement policy changes on hospital practices that perverse incentives may currently influence.

At the same time, available evidence on a possible phototherapy-autism link is already sufficient to support the application of the precautionary principle. Clinicians 50 years ago frequently advised mothers to switch their jaundiced, breastfed infants to formula. Then the pendulum swung in favor of the modern exclusive breastfeeding paradigm - a counter-intuitively medicalizing shift, backed by the naturalistic fallacy, that included phototherapy for the epidemic of jaundice associated with insufficient milk intake that it may have created. The evidence suggests it is past time it swung back.

Discussion

Meta-analytical estimates of increased possible autism risk associated with neonatal jaundice are consistently substantial. Their notably overlapping confidence intervals, based on included studies’ pooled ORs, reiterate estimated possible risk increases for near- and full-terms up to 67%, 68%, and 76%. These are sizeable possible effects.

Discrepant publication bias findings from previous meta-analyses reveal universal misuse of the funnel plot test, leaving open the question of whether selective results reporting may account for the jaundice-autism association. P-curve analysis finds that studies that estimate a statistically significant jaundice-autism link display no evidence of publication bias. We should think of this result as a quality control stamp supporting the validity of the widely-noted correlation.

Future research might extend this form of publication bias testing to the available evidence on complications of insufficient milk intake, filling a hole in the meta-analytical literature on the risk of preventable harm associated with common breastfeeding insufficiencies by evaluating a matrix of associated independent variables including hypoglycemia and hypernatremia, and dependent variables with which they covary according to systematic literature review. For jaundice, Amin et al suggested reconceptualizing the dependent variable in terms of a jaundice-associated subtle kernicterus or BIND spectrum of disorders including cognitive delay, ADHD, autism, specific learning disorder, and language disorder. A reconceptualized continuum might also include other manifestations of potential bilirubin-induced neurological harm such as cerebral palsy, epilepsy, hearing impairment, kernicterus, mood disorders, and lower IQ. It should also account for survivorship bias, especially in the preterm subgroup, by including death on a reconceptualized jaundice-associated harm continuum. This measure should count death from acute bilirubin encephalopathy (ABE) as well as death with an uncertain relationship to jaundice, particularly in less well-resourced settings.

Including death on the jaundice-associated harm continuum presents special challenges, because the cause of death in these settings may be difficult or impossible to establish. Acknowledging likely bias valence and magnitude, the ethical and practical significance of persistent racial and geographic disparities, and uncertainty may be the best we can do with available data; it is worth noting that here is uncertainty again, and writing it out of analyses leaves out the most vulnerable people concerned. This sort of limitation highlights again the importance of incorporating context, variety, and uncertainty into thinking about risk, and interpreting fuller findings rather than binary results. Synthesized results of the most current, correct, and comprehensive matrix of possible publication bias tests would advance knowledge about the evidential value of statistically significant links between complications of common breastfeeding insufficiencies and a spectrum of clinically significant harms. But that evidence is itself shaped by identified biases in practically significant ways that should at least be mentioned in its interpretation. Just because we cannot count sub-Saharan African infant deaths like we can WEIRD autism cases does not justify ignoring the former.

This dilemma resonates with Feinstein's concern that his evidence-based medicine revolution had been hijacked by the "distraction" of quantitative models [81]. He suggested differentiating between the special collection of data regarded as suitable evidence, and practicing evidence-based medicine [82]. Such data, he noted, derive "almost exclusively from randomized trials and meta-analyses," and "do not include many types of treatments or patients seen in clinical practice." Feinstein warned that by calling on doctors to make clinical decisions based on a highly restricted quality and scope of evidence, such work had an "authoritative aura" that "may lead to major abuses that produce inappropriate guidelines or doctrinaire dogmas for clinical practice." Kujabi et al.'s dismissal of the jaundice-autism link and relevant preventive measures based on their highly restricted and misinterpreted meta-analysis embodies this danger; but the entire exclusive breastfeeding paradigm is based on a large body of research that ignores serious neonatal risks, women's breastfeeding experiences, and the practical implications of major geographic disparities in healthcare access.

Thus Feinstein's warning also resonates more broadly with pro-breastfeeding bias, particularly confirmation bias, in the medical and public health literature [253]. Future research should critically review the relevant meta-analytical literature in terms of this bias. To what extent does it examine possible benefits to the exclusion of possible risks, tipping the scales in favor of a particular policy agenda before weighing the evidence? What role does the naturalistic fallacy play in framing the novel modern intervention of exclusive breastfeeding as effectively risk-free? How do cognitive and cultural biases like these interact with statistical methodological ones, as in Greenland's observation that standard categorical analysis neglects to account for within-category information [254] - a criticism most obviously relevant to the fact that breastfeeding works sooner and later, more and less well, for various reasons, for different mothers and infants, but is often treated as a binary variable in analyses? And what does the evidence suggest after considering potential confounds for exclusive breastfeeding's purported infant health benefits including established ones, chiefly maternal education and socio-economic status [182,255,256], as well as under-studied ones, chiefly breastfeeding insufficiencies [26] and maternal health? Or is there really such a poor evidence base in women's health that the data do not exist to answer this question?

Reapplying Feinstein's insight to the core finding of substantial possible jaundice-associated autism risk, this review notes that the meta-analytical literature may offer substantial underestimates. This article identifies numerous features in the jaundice-autism literature likely contributing to underestimation of associated harm. In addition to the features identified above - primarily effect dilution from mild case inclusion; selection against severe cases in study-level exclusion criteria and survivorship bias; underpowering from small sample size, low base rate, and over-narrow operationalization of the dependent variable as autism to the exclusion of other harms; and over-representation of WEIRD populations - there are two additional sources of possible underestimation bias that bear mentioning.

Operationalizing jaundice as TSB concentration (at best) is another way in which the literature may tend to underestimate the jaundice-autism effect. TSB reportedly has high sensitivity but low specificity for associated brain injury in near- and full-term infants [28], so relying on it may dilute the effect by including a large number of so-called "false positives," insofar as TSB could be an imperfect proxy for an underlying jaundice-associated risk factor or factors. Measuring unbound unconjugated bilirubin (UB) is more predictive but more technically difficult, and so unlikely to become part of routine care, particularly in less well-resourced settings that disproportionately bear the burden of associated harm [257]. And, as with elevated TSB, there is no established safe level of elevated UB vis-a-vis neurodevelopmental harm. Future research using UB in better-resourced settings might be feasible. But this shift to a more precise measurement runs the risk of focusing on harm once irreversible damage is done, rather than preventing it; if substantial risk is uncertain but possible, and damage may be done by the time of diagnosis, then preventing and promptly treating jaundice to prevent bilirubin-induced neurotoxicity should be the primary focus in research and practice. In that context, simpler, cheaper, and less invasive metrics (such as weight loss and waste outputs) make more sense than relatively costly and invasive ones, and harm prevention protocols (such as default prelacteal feeding) make more sense than either for improving patient outcomes. The logic of these alternatives is particularly persuasive when we remember that jaundice itself may proxy for insufficient milk intake, which could cause irreversible neurological damage along multiple pathways.

Relatedly, interaction presents an additional source of possible underestimation and overestimation bias; other predisposing factors and associated conditions may interact with jaundice to magnify risk in many groups, and this magnification may be part of what existing jaundice- and phototherapy-autism effect estimates are really capturing. Subgroup effects (e.g., in infants with infection and/or birth trauma, and racial subgroups with heightened genetic vulnerabilities - notably including traditionally disadvantaged groups) might be difficult to estimate using existing datasets; as previously discussed, there are theoretical and empirical reasons for clinicians to have a lower threshold of suspicion for neonatal jaundice and associated risk in many subgroups. Such clinically relevant subgroup effects may include and interact with etiological subgroup effects. In particular, jaundice associated with insufficient milk intake from breastfeeding correlates in a non-negligible percentage of cases with other complications of insufficient milk intake, chiefly hypernatremic dehydration and hypoglycemia. These other complications could matter for neurodevelopmental risk. While future research might better quantify these conditions' overlap and relationships with risk, a harm prevention focus better applies fundamental medical and research ethics than an approach that focuses primarily on better measuring irreversible damage once it has been done.

At the same time, this identification of numerous likely underestimation biases underscores the importance of taking seriously consistent meta-analytic findings of a substantial possible jaundice-autism link. That these findings are consistent is a novel insight: This review shows that the article by Kujabi et al. contains mistakes that, if corrected, would support different conclusions. Its authors misinterpreted statistical significance test results to claim no jaundice-autism association when their analysis showed a quite sizeable possible effect. They also misused the funnel-plot test for publication bias, claiming without merit that its results indicated a high risk of publication bias. Their claim that "there is no evidence to suggest jaundice should be treated more aggressively to prevent autism" was based on these mistakes, incorrect temporal sequencing, and apparent lack of consideration of evidence on jaundice severity, etiology, and different prevention, screening, and treatment approaches. Such mistaken conclusions are likely to contribute to practices that endanger neonates. Kujabi et al. did not substantively respond to attempts to communicate about these issues. The relevant journal, Pediatric Research, declined to retract the paper - despite a six-month lag between this author's first manuscript submission raising these concerns (May 2021) and the journal's print publication of Kujabi et al.'s meta-analysis (November 2021). The journal also declined to publish earlier versions of this review, which was revised and resubmitted at the journal's direction as an article and letter to the editor. As stated in correspondence with the journal, failing to address these issues may contravene relevant publishing ethics [258] and retraction guidelines [259], which emphasize the importance of protecting the integrity of the scientific record. Thus in the absence of other recourse, this article protects the integrity of the literature by correcting these mistakes, so the scientific record accurately reflects the empirical basis of an urgently needed shift. Medical practitioners should be aware of the evidence linking jaundice with autism, and act to prevent jaundice progression and possibly associated harm.

The evidence supports a paradigm shift in early infant feeding that is radical from the contemporary perspective, but that only represents a long-overdue pendulum swing back toward previous norms. Modern early infant feeding emphasis on exclusive breastfeeding breaks with all known, prior such practices by introducing a days-long neonatal starvation period that compounds the risks associated with the historically anomalous structural vulnerability of a single point of failure, one mother as the exclusive source of recommended infant nutrition and hydration. This break corresponds with a substantial increase in reported autism prevalence that may be causally explained, at least in part, by a corresponding increase in brain damage from increased neonatal jaundice associated with insufficient milk intake due to common breastfeeding insufficiencies as the new exclusive breastfeeding paradigm achieved ascendance.

If previously healthy full-term neonates are the experimental group in this natural experiment, preterms are the control group, as they are typically supplemented early and often. The preterm paradox of weakened jaundice-autism effect estimates in this subgroup is consistent with a protective effect of this supplementation. The three previous meta-analyses (Amin et al., Jenabi et al., and Kujabi et al.) agree that the evidence for a jaundice-autism link appears weaker among preterms, losing statistical significance as well as magnitude, and generating estimated possible risk increase values up to 2%, 13%, and 12%, respectively. This weakened effect might be explained in part by factors relating to the "experiment" and in part by other factors. Crucially, heightened monitoring combined with more supplementation likely reduces preterm starvation periods and associated risks (e.g., failure to clear bilirubin as waste) in comparison with full-terms, protecting their brains from bilirubin-induced neurodevelopmental harm. Unrelatedly, the effect could also appear (but not necessarily be) weaker due to smaller subgroup sample sizes, survivorship bias as more preterms die leaving fewer to develop autism, and relatively greater full-term remaining susceptibility with respect to a triggering threshold of cumulative adverse event exposure.

Safety concerns about phototherapy are similarly consistent with an overarching story about under-recognized risks associated with the exclusive breastfeeding paradigm, and the crisis in science that they illustrate. Included studies that analyze a possible phototherapy-autism link tend to suggest a possible substantial increased risk of even greater magnitude than the possible jaundice-autism link. A common misuse of statistical significance testing again plays a role in the under-recognition of this risk. Until more definitive studies are available, medical practitioners should try to prevent autism and other possible long-term complications of jaundice and phototherapy treatment. This means that neonatal jaundice needs more aggressive - but less medicalizing - prevention, diagnosis, and treatment. Early, adequate, and often formula or other appropriate infant milk supplementation would prevent harm from insufficient milk intake associated with breastfeeding, the most frequent cause of jaundice in previously healthy near- and full-term neonates. At least until more evidence establishes phototherapy as safe, such supplementation should replace it as the standard of care, ideally before jaundice is even diagnosed in breastfed infants; and when phototherapy treatment is still deemed medically necessary, complementary supplementation should also be standard of care due to greater bilirubin clearance in formula-fed versus breastfed neonates.

Future research should investigate how this shift affects neonates with respect to jaundice, later neurodevelopmental outcomes, other potentially associated outcomes such as allergic diseases, and mortality in a variety of settings. How might that research be structured? This question matters for meta-analysis; current methodological recommendations for systematic reviews and meta-analyses, in the form of the Cochrane Handbook chapter on assessing the risk-of-bias in a non-randomized study, suggest authors start by describing "a 'target trial', which is a hypothetical pragmatic randomized trial of the interventions compared in the study, conducted on the same participant group and without features putting it at risk of bias" [72]. One such trial might involve a cluster-randomized experiment assigning a diverse group of hospitals to transition sooner (treatment) or later (control) to the prelacteal feeding alternative to the modern exclusive breastfeeding paradigm. This would give hospitals a chance to update related prenatal/parental as well as staff educational content to reflect the evidence supporting these practices, so that primary caregivers and medical practitioners alike understand the importance of keeping neonates adequately fed (i.e., preventing death or permanent brain damage), the safety profile of formula-feeding in relevant contexts (i.e., excellent wherever water is clean, literacy high, and formula access stable), and ways in which keeping neonates fed need not conflict with supporting breastfeeding (i.e., there is insufficient evidence to establish a forced choice between supporting breastfeeding and feeding hungry infants). A second study arm involving random assignment to phototherapy for a subgroup of jaundiced neonates (neither so mildly jaundiced that they would not generally be said to require treatment, nor so severely jaundiced that it would be considered unethical to withhold current standard care). These two study arms would make a 2x2 design permitting analysis of the interaction between formula supplementation and phototherapy. Hospitals could thus implement new (instantiations of old) early infant feeding policies prioritizing harm prevention (i.e., ensuring neonates receive sufficient early nutrition/hydration by supplementing early and often with adequate formula), and then analyze how the change affects jaundice and associated morbidity/mortality after the fact (e.g., hypothesizing substantial reductions in preventable neonatal readmissions and later neurodevelopmental disorders including autism), including possible iatrogenesis from phototherapy as distinct from the effects of insufficient milk intake.

In line with the Cochrane Handbook guidance, this target trial suggests direction for future meta-analysis. Are formula-fed neonates less prone to phototherapy-associated risks, correcting for possible confounds including prematurity and jaundice severity/duration? Do available data let us see whether other complications commonly associated with insufficient milk intake, like hypoglycemia, seem to be associated with phototherapy risk, which might support the hypothesis that phototherapy proxies for starvation? What do data from jaundice complication prevention experiments show?

In sum, meta-analyses consistently estimate a quite substantial possible neonatal jaundice-autism effect. The association seems real; there is no evidence of publication bias. Severity and time matter, so prevention is key. Racial and geographic disparities are troubling, and there are many likely sources of underestimation bias in existing estimates including such disparities. The introduction and rise of the historically anomalous exclusive breastfeeding paradigm coincide with and could causally explain increasing autism prevalence from brain damage from complications of insufficient milk intake including jaundice. Preterms' weaker jaundice-autism link is consistent with strong supplementation norms protecting that subgroup. An even more substantial possible phototherapy-autism link is consistent with untreated insufficient milk intake doing at least part of the damage. This review synthesizes the state of the literature, which contains clear and convincing evidence that jaundice may be associated with common and preventable harm to neonates. So what is the public health apparatus doing to protect them?

Documents obtained recently through the Freedom of Information Act (FOIA) appear to show that US Centers for Disease Control and Prevention (CDC) awareness of exclusive breastfeeding-associated risks, including the possible jaundice-autism link, has outpaced related safety monitoring and harm prevention efforts [260]. A FOIA request sought to obtain records on any CDC "actions to prevent disease, injury, and disability associated with risks of insufficient newborn milk intake under the exclusive breastfeeding policies and practices the CDC promotes," as well as on CDC responses to or about the grassroots, non-profit Fed Is Best Foundation's efforts to raise public awareness of exclusive breastfeeding risks, and to prevent associated harm, and to or about Fed Is Best co-founder Christie del Castillo-Hegyi. Disclosed documents reveal no relevant safety monitoring or harm prevention efforts. They indicate that the agency is aware of evidence linking exclusive breastfeeding with jaundice, and jaundice with autism risk. They show that, in spite of this awareness, the CDC has recently continued promoting and funding the implementation of the exclusive breastfeeding paradigm, including communicating at the highest levels with exclusive breastfeeding proponents like the Baby-Friendly USA organization and other leading breastfeeding activist organizations and individuals on meeting agendas, public campaigns, and responses to Fed Is Best's efforts. Some of these responses, about which the CDC was made aware, deny without evidence the merits of the Foundation's claims that exclusive breastfeeding commonly risks preventable harm to neonates. But the medical literature supports these claims [26].

The CDC's combination of inaction in public health surveillance on one hand, and continued action in exclusive breastfeeding funding and promotion on the other, contributes substantially to the ongoing dominance of the exclusive breastfeeding paradigm in the US, which may contribute to the paradigm's continued global dominance. Heightened democratic oversight might enhance CDC efforts to fulfill its functions including preventing harm to neonates. For instance, the apparent lack of exclusive breastfeeding safety monitoring and harm prevention efforts, despite agency knowledge of associated risks, might be an appropriate subject for a Congressional inquiry, Government Accountability Office (GAO) review, class action lawsuit, or other standard democratic action intended to bring actual public agency behavior into greater accord with stated public agency goals and obligations.

## Conclusions

Neonatal jaundice may substantially elevate the risk of preventable, irreversible neurological damage including autism. Publication bias testing with p-curve analysis finds that studies that estimate a statistically significant jaundice-autism link do not show evidence of selective results reporting; they have evidential value. Jaundice severity heightens risk, so preventing progression is key; in most cases, it is as easy as giving a bottle. Racial and geographic disparities seem to mean that traditionally disadvantaged groups are worst-affected by jaundice-associated harm, and global unrepresentativeness likely generates considerable underestimates of associated harm including death and permanent disability. Additional identified factors likely further bias available estimates down, particularly overly narrow operationalization of the outcome of interest, survivorship bias, and inclusion/exclusion criteria that tend to include mild cases and exclude severe ones. The preterm paradox, in which a more vulnerable subgroup appears less vulnerable to the jaundice-autism link, may be due largely to different early infant feeding norms that minimize preterm deprivation/starvation; preterms may be the control group in the exclusive breastfeeding natural experiment. Phototherapy, the current standard treatment for neonatal jaundice, may additionally increase autism risk but could proxy for untreated insufficient milk intake.

This review synthesizes a large body of evidence that strongly supports precautionary principle invocation. Until more definitive studies are available, medical practitioners should try to prevent autism and other possible long-term complications of neonatal jaundice by treating its modal root cause, insufficient milk intake associated with breastfeeding. Breastfeeding need not confer risk of preventable neurodevelopmental harm. It is rather the modern construction of "exclusive breastfeeding" - a practice without historical precedent that was invented relatively recently by reformers lacking intergenerational knowledge about breastfeeding safety - that introduces this risk by omitting the safety infrastructure that characterized prior societies' early infant feeding practices; prelacteal feeding, shared nursing, and wetnursing guarded against harm from common breastfeeding insufficiencies. Mothers' milk usually takes days to come in, days in which newborns in previous advanced civilizations (and foraging societies today) would usually drink other mothers' milk or another substitute; ours may be the only society ever in human history in which experts routinely advise caretakers to deprive newborns of adequate nutrition and hydration for days. A return to previously widespread prelacteal and other supplementary feeding practices characterized by early, adequate, and often formula (or other appropriate milk) supplementation would prevent harm from insufficient milk intake associated with breastfeeding, the modal cause of jaundice in previously healthy near- and full-term neonates. Future research and practice should apply the precautionary principle to act to avoid harm when risks are uncertain and stakes are high. Early infant feeding and jaundice management guidelines urgently require change.
